# CREB1 promotes expression of immune checkpoint HLA-E leading to immune escape in multiple myeloma

**DOI:** 10.1038/s41375-024-02303-w

**Published:** 2024-06-20

**Authors:** Aya Ismael, Allen J. Robinette, Laila Huric, Jamie Schuetz, Kameron Dona, Don Benson, Emanuele Cocucci, Francesca Cottini

**Affiliations:** 1 Division of Hematology, Department of Internal Medicine, The Ohio State University College of Medicine, Columbus, OH USA; 2 Comparative Pathology and Digital Imaging Shared Resource Main Laboratory, The Ohio State University College of Veterinary Medicine, Columbus, OH USA; 3 Division of Pharmaceutics and Pharmacology, The Ohio State University College of Pharmacy, Columbus, OH USA

**Keywords:** Immunosurveillance, Myeloma

## Abstract

Multiple myeloma (MM) cells effectively escape anti-tumoral immunity to survive in the tumor microenvironment (TME). Herein, we identify non-classical major histocompatibility complex (MHC) class I molecule HLA-E as a major contributing factor in immune escape. Clinically, HLA-E expression correlates with aggressive disease features such as t(4;14) and CD56 expression and is induced by IFN-gamma (IFN-γ) in the TME. We discovered that HLA-E is regulated by cAMP responsive element binding protein 1 (CREB1) transcription factor by direct promoter binding; genomic and pharmacological inhibition of CREB1 reduced HLA-E levels even in the presence of IFN-γ or IFN-γ activating agents, such as immunomodulatory drugs and panobinostat. HLA-E binds to natural killer group 2A (NKG2A), delivering an inhibitor signal to natural killer (NK) cells. Treatment with a CREB1 inhibitor was able to restore NK cell-mediated cytotoxicity against MM cell lines and patient samples. In conclusion, our results strongly demonstrate that CREB1 inhibition promotes anti-tumoral immunity in MM by limiting HLA-E expression and enhancing the activity of NK cells.

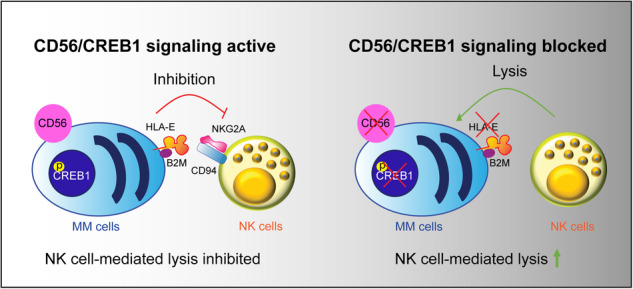

## Introduction

Multiple Myeloma is a disease of clonal aberrant plasma cells, which accumulate in the bone marrow (BM) and interact with the tumor microenvironment (TME) to thrive. Despite more than 20 drugs approved, MM is still incurable, and patients inevitably experience relapse of their disease and develop toxicities and functional decline [1]. Active suppression of anti-tumor immune responses or recruitment of anti-inflammatory immune cells, such as exhausted or anergic T cells or inhibitory natural killer (NK) cells, lead to immune escape and reduced response to immunomodulatory drugs (IMiDs), T-cell engagers (TCE), and chimeric antigen receptor (CAR) T-cell therapies. NK cells have an innate ability to detect and kill cancerous cells [2] and their presence in the TME is usually a good prognostic feature in hematological malignancies [3]. However, patients with MM have significant changes in the distribution, phenotype, and functional activity of NK cells [4–6], leading to reduced cytotoxicity.

How MM cells drive immune escape, even in the absence of selective pressure from therapy, is vastly unknown. We hypothesize that HLA-E could be involved in this process. HLA-E (major histocompatibility complex, class I, E) is a non-classical major histocompatibility complex (MHC) class I molecule [7], which binds to natural killer group 2A (NKG2A)^+^, CD94^+^ T cells and NK cells, leading to immune escape [8]. HLA-E can also bind to NKG2C/D, having opposite immune stimulating effects [9]. HLA-E expression confers poor prognosis in MM [10]. Moreover, specific subsets of patients with MM, such as those with t(4;14), have higher expression of HLA-E [11].

Herein, we elucidate the function and regulation of HLA-E in MM and investigate strategies to reduce its expression and improve NK cell-mediated cytotoxicity. We demonstrate that HLA-E expression is present in pre-malignant plasma cell disorders (monoclonal gammopathy of undetermined significance – MGUS and smoldering multiple myeloma – SMM) and in overt MM. Using gain-of- and loss-of-function models, ChIP-sequencing, RNA-sequencing, and protein analysis we describe for the first time that cAMP responsive element binding protein 1 (CREB1) regulates STAT1 and HLA-E. The inhibition of CREB1 reduced the survival of MM cells and promoted NK cell-mediated cytotoxicity as a single agent or in combination with immunomodulatory drugs. These findings highlight the role of CREB1 in immune escape, leading to novel therapeutic approaches for treating patients with MM.

## Materials and methods

### Cell lines and cultures

The human cell lines H929, RPMI-8226, MM.1S, MM.1R, and HEK293T cells were purchased from the American Type Culture Collection (ATCC), while the human cell lines U266 and OPM-2 were purchased from the Leibniz Institute DSMZ – German Collection of Microorganisms and Cell Cultures (DSMZ). Human cell lines KMS-11 and KMS-11 BTZ were kindly provided by Dr. Arianna Giacomini, University of Brescia, Italy. All MM cell lines (H929, RPMI-8226, MM.1S, MM.1R, U266, OPM-2, KMS-11, and KMS-11 BTZ) were cultured in RPMI-1640 (Gibco, Thermo Fisher Scientific, Waltham, MA, USA) medium containing 10% fetal bovine serum (FBS, Gibco, Thermo Fisher Scientific), 2 µM/L glutamine, 10,000 U/mL penicillin G, and 10,000 μg/mL streptomycin (Gibco, Thermo Fisher Scientific) and maintained at 37 °C with 5% CO_2_. HEK293T cells were cultured in Dulbecco’s Modified Eagle Medium (DMEM) (Gibco, Thermo Fisher Scientific), containing 10% FBS, 2 µM/L glutamine, 10,000 U/mL penicillin G, and 10,000 μg/mL streptomycin and maintained at 37 °C with 5% CO_2_. Cells were used within 2–3 months after thawing (24–40 passages at maximum). Cell lines were also regularly tested for mycoplasma contamination using the MycoAlert mycoplasma detection kit (Lonza, Basel, Switzerland).

### MM patient samples

Bone marrow (BM) aspirates were obtained under Institutional Review Board (IRB)-approved protocols (2010C0126 and 2021C0218) after informed consent in accordance with the Declaration of Helsinki was obtained from all subjects. MM cells were separated by Ficoll-Paque (Cytiva, Cat. No. 17144002) suspension, followed by CD138 positive selection, using EasySep™ Human CD138 Positive Selection Kit II (RosetteSep™, STEMCELL Technologies, Cat. No. 17877). Purity was confirmed by CD138-V450 (BD Biosciences, San Jose, CA, USA, Cat. No. 562098, RRID:AB_10894011) and CD38-PE (BD Biosciences, Cat. No. 555460, Clone HIT2, RRID:AB_395853) staining. Peripheral blood (PB) samples were separated by density gradient centrifugation over Ficoll-Paque, washed with centrifugation with room-temperature phosphate-buffered saline (PBS, Gibco), and stained with specific flow cytometry fluorescent antibodies.

### NK cell isolation by RosetteSep™ immunodensity cell separation method

Apheresis cones were obtained from Versiti, Inc. After dilution of full apheresis blood in a 50 mL tube with PBS, 500 µL of RosetteSep™ Human NK Cell Enrichment Cocktail (STEMCELL Technologies, Cat. No. 15065) were added. Blood was incubated for 20 min at room temperature on a rocker. 15 mL of Ficoll solution was added to a new tube and topped with 35 mL of blood. The sample was centrifuged at 1800 rpm for 30 min at room temperature. Buffy layers were collected, and cells were washed twice in PBS (1300 rpm for 5 min). Ammonium chloride solution (STEMCELL Technologies, Cat. No. 07850) for RBC lysis was added to the pellet and incubated at room temperature for 5 min. Cells were washed in PBS, counted, and stained with specific flow cytometry fluorescent antibodies.

### RNA extraction and quantitative real-time PCR analysis

RNA for quantitative real-time PCR was extracted using TRIzol method (Invitrogen, Life Technologies, Carlsbad, CA, USA) or ReliaPrep™ RNA Miniprep System (Promega, Madison, WI, USA, Cat. No. Z6011); cDNA was synthesized using the ProtoScript® II First Strand cDNA Synthesis Kit (New England Biolabs, Ipswich, MA, USA). Quantitative real-time PCR analysis was performed using SYBR GREEN PCR Master Mix protocol (Applied Biosystems, CA, USA, Cat. No. 4309155). Quantitative real-time PCR analysis was performed on a ViiA 7 Real-Time PCR System (Applied Biosystems, CA, USA). Data were analyzed using the ΔΔ Ct method. Gene-specific primers are reported in the Supplementary Methods. GAPDH was used for normalization.

### Western blot analysis

MM cells were harvested and lysed using RIPA lysis buffer (Cell signaling, Cat. No. 9806), with addition of 1 mM PMSF (Cell signaling, Cat. No. 8553). Laemmli sample buffer (BIO-RAD, Cat. No. 1610747) was added and samples were boiled at 95 °C. Cell lysates were subjected to SDS–PAGE, transferred to nitrocellulose membranes, and immunoblotted with the specific antibodies reported in the Supplementary Methods. All antibodies were diluted to a concentration of 1:1000 and prepared in milk 5% diluted in TBS (BIO-RAD, Cat. No. 1706435) with Tween 20 (BIO-RAD, Cat. No. 1706531).

### Gain-of- and loss-of-function experiments in MM cell lines

For gain-of-function experiments, U266 cells were transiently transfected using Nucleofector 4D Unit X, Kit SF, program DY-100 (Lonza, Amaxa Biosystems, Köln, Germany). Viruses were produced by co-transfection of specific plasmids and packaging vectors (psPAX2, Addgene Cat. No. 12260, RRID:Addgene_12260 and pMD2.G, Addgene Cat. No. 12259, RRID:Addgene_12259) into HEK293T cells. H929 and OPM-2 cells were infected with lentiviral particles using polybrene followed by spinoculation of suspended cells. Stably infected cells were selected using 0.5–1 μg/mL of puromycin (InvivoGen, Cat. No. ant-pr-1). After transfection or infection, MM cells were subjected to mRNA analysis, western blotting, and flow cytometry analyses.

### Flow cytometry analysis

MM cell lines, MM patient-derived samples, and PB samples were washed with room-temperature PBS and then incubated with specific antibodies for 20 min. Cells were then washed with PBS and acquired on Attune NxT Flow cytometry machine using Attune NxT Software v 3.1 (Thermo Fisher Scientific). At least 50,000 events were acquired. The following antibodies were used: HLA-E-PE (BioLegend, San Diego, CA, USA, Cat. No. 342604), CD56-APC (BD Biosciences, Cat. No. 555518, RRID:AB_398601), CD56-PE (Miltenyi Biotec, Cat. No. 170-081-014, Clone REA196), CD138-V450 (BD Biosciences, Cat. No. 562098, RRID:AB_10894011), and CD38-PE (BD Biosciences, Cat. No. 555460, Clone HIT2, RRID:AB_395853). For staining of NK cell populations in the peripheral blood, the following antibodies were used: CD3-VioBlue (Miltenyi Biotec, Cat. No. 170-081-046, Clone BW264/56), CD16-FITC (BD Biosciences, Cat. No. 555406, Clone 3G8), CD56-APC Vio770 (Miltenyi Biotec, Cat. No. 130-114-548, Clone REA196), CD94-PE (BD Biosciences, Cat. No. 555889, Clone HP-3D9, RRID:AB_396201), NKG2A/CD159a-APC (Miltenyi Biotec, Cat. No. 130-113-563, Clone REA110). Post-acquisition analyses including t-SNE analysis were performed using FlowJo Software (BD Biosciences).

### Co-culture experiments of NK cells with MM cells

MM cells were modified by gain-of- or loss-of-function approaches or treated with different compounds (day 1) for 48 h in a six-well plate. On day 2, apheresis cones from healthy donors were shipped overnight in dry ice from Versiti, Inc. On day 3, NK cells were isolated by RosetteSep™ immunodensity cell separation method as described above. Both MM and NK cells were counted and plated at an effector to target (E:T) ratio of 5:1 or 10:1 in V-shaped 96-well plates. After 4 h, the cells were stained with CD138-V450 (BD Biosciences, Cat. No. 562098, RRID:AB_10894011), CD56-PE (Miltenyi Biotec, Cat. No. 170-081-014, Clone REA196), CD16-FITC (BD Biosciences, Cat. No. 555406, Clone 3G8), and SYTOX™ Red Dead Cell Stain (Thermo Fisher Scientific, Cat. No. S34859) and acquired on Attune NxT Flow cytometry machine. MM cells without NK cells were plated, stained, and acquired in the same conditions. The percentage of lysis was calculated normalizing to the average of *n* = 3 treated cells without NK cells.

### Analysis of RNA-sequencing or gene-expression profiling from multiple myeloma datasets

The RNA-sequencing data were downloaded from the MMRF CoMMpass database, with data generated as part of the Multiple Myeloma Research Foundation Personalized Medicine Initiatives (https://research.themmrf.org and www.themmrf.org). mRNA expression data were collected from: GSE4452, a collection of 12 healthy donors and 65 newly diagnosed MM patients; GSE5900, a collection of 22 cases of healthy donors, 44 cases of MGUS, and 12 cases of SMM; and GSE8546, a collection of relapsed patients with MM prior to (*n* = 36) and after (*n* = 19) lenalidomide administration. For GSE8546, 16 samples were paired and used for the analysis. All three datasets were analyzed by Affymetrix microarray (HG-U133 Plus 2.0 array for GSE4452 and GSE5900; HG U95Av2 array for GSE8546). For evaluation of the relationship between HLA-E, STAT1, IRF1, and IRF9, with CREB1 or CD56, median value of expression was used as threshold to define two groups. Gene sets of Canonical Pathways (C2_CP_BIOCARTA, C2_CP_KEGG, C2_CP_PID, C2_CP_REACTOME, C2_CP_WIKIPATHWAYS) or gene ontology (C5_GOBP) were used to perform enrichment analysis at http://www.broad.mit.edu/gsea [12, 13]. Two-sided *p* values < 0.05 were considered statistically significant. Statistical analyses were performed using GraphPad software (GraphPad Prism, RRID:SCR_002798).

## Results

### HLA-E is expressed by MM cell lines and patients

**Fig. 1:**
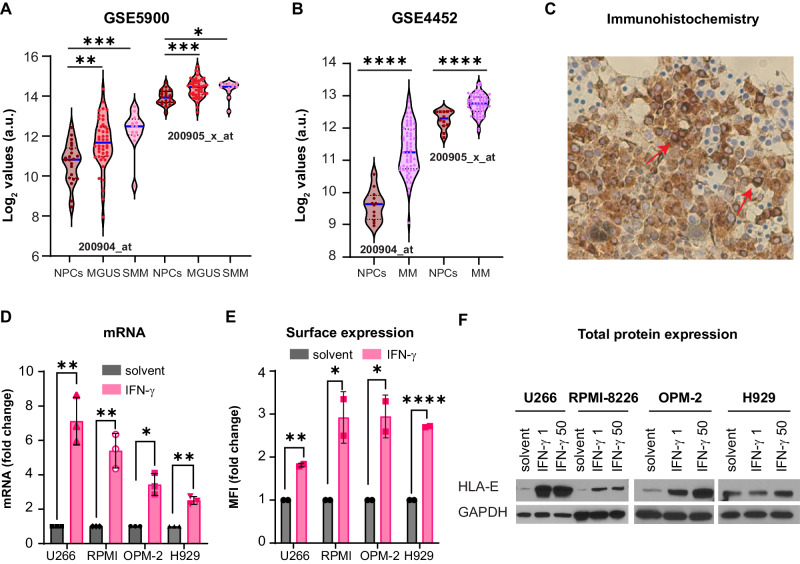
HLA-E is expressed in MM cells and induced by IFN-γ. **A** HLA-E log_2_ mRNA expression values in bone marrow plasma cells from healthy individuals (NPCs, *n* = 22), individuals with monoclonal gammopathy of undetermined significance (MGUS, *n* = 44), or individuals with smoldering multiple myeloma (SMM, n= 12). 2000904_at probe: ANOVA *p* value = 0.0004; NPCs vs MGUS *p* = 0.0036, **; NPCs vs SMM *p* = 0.0005, ***. 200905_x_at probe: ANOVA *p* value = 0.0005; NPCs vs MGUS *p* = 0.0003, ***; NPCs vs SMM *p* = 0.0179, *. Solid blue lines indicate the median values; black dotted lines represent the 25^th^ and 75^th^ percentile. Data are derived from GSE5900 dataset. **B** HLA-E log_2_ mRNA expression values in bone marrow plasma cells from healthy individuals (NPCs, *n* = 12) or patients with MM (MM, *n* = 65); 2000904_a probe*: p* < 0.0001, ****; 200905_x_at probe: *p* < 0.0001, ****. Solid blue lines indicate the median values; black dotted lines represent the 25^th^ and 75^th^ percentile. Data are derived from GSE4452 dataset. **C** Representative immunohistochemistry staining for HLA-E (20×) in one newly diagnosed patient with MM. Red arrows show MM cells positive for HLA-E. **D** HLA-E mRNA fold change in a panel of MM cell lines (U266, RPMI-8226, OPM-2, and H929) treated with solvent or IFN-γ 1 ng/mL for 24 h. *n* = 3, t test, two-tailed; U266 *p* = 0.0096, **; RPMI-8226 *p* = 0.0016, **; OPM-2 *p* = 0.0141, *; H929 *p* = 0.0031, **. Treated cells are normalized to each control. **E** HLA-E mean fluorescence intensity (MFI) fold change in a panel of MM cell lines (U266, RPMI-8226, OPM-2, and H929) treated with solvent or IFN-γ 1 ng/mL for 24 h. *n* = 2, t test, two-tailed; U266 *p* = 0.0014, **; RPMI-8226 *p* = 0.0455, *; OPM-2 *p* = 0.0311, *; H929 *p* < 0.0001, ****. Treated cells are normalized to each control. **F** Western blot analysis for HLA-E and GAPDH in a panel of MM cell lines (U266, RPMI-8226, OPM-2, and H929) treated with solvent, IFN-γ 1 ng/mL, or IFN-γ 50 ng/mL for 24 h.

To study the role of HLA-E in MM, we first analyzed mRNA gene-expression data from healthy donors, individuals with monoclonal gammopathy of undetermined significance (MGUS), smoldering MM (SMM), and MM in publicly available datasets, such as GSE4452 and GSE5900. HLA-E mRNA expression was higher in MGUS, SMM, and MM cells compared with normal plasma cells (NPCs) (Fig. 1A, B), suggesting it is an early myelomagenesis event. We then confirmed the presence of HLA-E protein expression by flow cytometry in MM patient samples and MM cell lines (Fig. S1A, B) and by immunohistochemistry in newly diagnosed patients (Fig. 1C and Fig. S1C). IFN-γ, a known regulator of HLA-E expression [14], is abundant in the BM-TME where MM cells reside, and its levels increase with disease progression [15]. Treatment of MM cell lines with IFN-γ further augmented HLA-E mRNA expression (Fig. 1D and Fig. S1D), the presence of HLA-E on the cell surface (Fig. 1E), and the total HLA-E protein levels (Fig. 1F and Fig. S1E). These data suggest that the presence of IFN-γ in the TME can modulate HLA-E expression, leading to higher HLA-E levels in patients, compared to in vitro conditions.

### HLA-E expression correlates with CD56 and CREB1 expression in patients

**Fig. 2:**
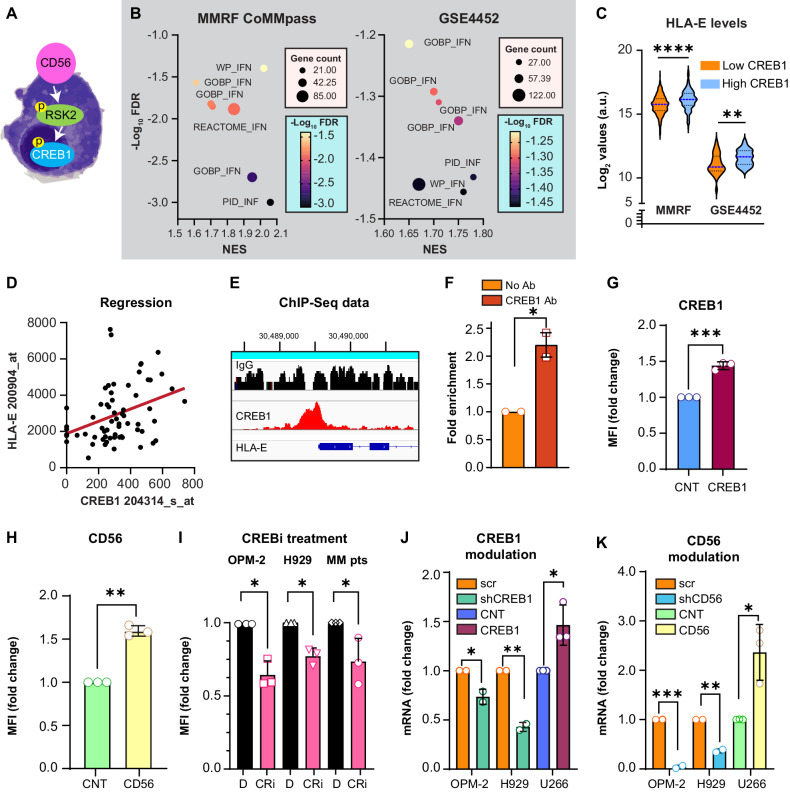
CD56 and CREB1 modulate HLA-E expression in MM. **A** Schema of CD56-CREB1 signaling in MM. CD56 induces phosphorylation of RPS6KA3 (also known as RSK2), which in turn phosphorylates CREB1, leading to gene transcription. **B** Pathway analysis of the upregulated IFN_driven gene sets in the MMRF CoMMpass and GSE4452 datasets. Patients are divided based on median cutoff of CREB1 expression. NES normalized enrichment score; FDR false discovery rate; gene count includes the number of significant genes in the pathway. **C** HLA-E log_2_ expression values in patients with low or high CREB1 expression based on median cutoff of CREB1 expression. CoMMpass MMRF dataset: *n* = 809, *p* < 0.0001, **** and GSE4452 dataset: *n* = 65, *p* = 0.0061, **. Dashed blue lines indicate the median values; black dotted lines represent the 25^th^ and 75^th^ percentile. **D** Regression studies to correlate HLA-E (probe 200904_at) as dependent variable to CREB1 (probe 204314_s_at). *p* = 0.0031; *R* = 0.13. **E** ChIP-sequencing tracks showing CREB1 signal on individual locus for HLA-E. The x-axis shows genomic coordinates. **F** Quantitative PCR of CREB1-ChIP enriched binding site to HLA-E promoter. *n* = 2, t test, two-tailed; *p* = 0.0317, *. **G** HLA-E MFI fold change in U266 cells overexpressing CREB1 compared with U266 control cells (CNT). *n* = 3, t test, two-tailed; *p* = 0.0001, ***. **H** HLA-E MFI fold change in U266 cells overexpressing CD56 compared with U266 control cells (CNT). *n* = 3, t test, two-tailed; *p* = 0.0010, **. **I** HLA-E MFI fold change in OPM-2 cells, H929 cells, and CD56^+^ CD138^+^ patient-derived MM cells treated with DMSO (D) or 666-15 (CRi) 0.3 μM for 48 h. *n* = 3, t test, two-tailed; OPM-2 *p* = 0.0153, *; H929 *p* = 0.04, *; MM patient samples (MM pts) *p* = 0.04, *. **J** HLA-E mRNA fold change in OPM-2 and H929 cells silenced for CREB1 (shCREB1) or with scrambled vectors, scr (*n* = 2, t test, two-tailed; *p* = 0.039, * and 0.0031, **) and in U266 cells overexpressing CREB1 or the control vector- CNT (*n* = 3, t test, two-tailed*; p* = 0.05, *). **K** HLA-E mRNA fold change in OPM-2 and H929 cells silenced for CD56 (shCD56) or with scrambled vectors, scr (*n* = 2, t test, two-tailed; *p* = 0.0008, *** and 0.0021, **) and in U266 cells overexpressing CD56 or the control vector-CNT (*n* = 3, t test, two-tailed; *p* = 0.04, *).

Patients with t(4;14), compared with patients with other chromosomal abnormalities, have larger MM cell clones that express CD56 (also known as neural cell adhesion molecule 1). In turn, CD56 activates the transcription factor CREB1, as shown in Cottini et al. [16], and in Fig. 2A. Interestingly, patients with CD56^positive^ MM also express HLA-E at higher levels [11] and have an immune inhibitory signature [17]. We therefore hypothesized that CREB1 could regulate HLA-E expression in MM. mRNA or RNA-sequencing data from patients with MM based on high and low CREB1 expression (*n* = 65, GSE4452 dataset, and *n* = 809, CoMMpass dataset) were then analyzed. In both the GSE4452 and CoMMpass datasets, gene set enrichment analysis (GSEA) showed increased expression of several pathways related to IFN-γ signaling in patients with high CREB1 expression (Fig. 2B and Fig. S2A, B), and in U266 cells overexpressing CD56 (Fig. S2C, D) or CREB1 (Fig. S2E). We then specifically demonstrated that patients with high CREB1 expression had statistically significant higher HLA-E levels compared with patients with low CREB1 expression (CoMMpass MMRF: *p* = 0.0015, **; GSE4452: *p* = 0.0013, **, Fig. 2C), with a positive correlation between CREB1 and HLA-E paired mRNA levels in the linear regression analysis (*p* = 0.0031, *R* = 0.13, Fig. 2D). ChIP-sequencing analysis in H929 cells (Fig. 2E) and fold enrichment analysis by RT-qPCR confirmed binding of CREB1 to HLA-E promoter (Fig. 2F) as predicted by in silico analysis from the Encode GM23338 dataset (Fig. S3A). Treatment with a specific inhibitor of CREB1, called 666-15 (CREBi), reduced binding of CREB1 to the HLA-E promoter (Fig. S3B). These in silico data suggested that CREB1 could influence HLA-E expression in MM.

### CREB1 modulates HLA-E expression even in the absence of IFN-γ

With the intent of confirming whether CREB1 was truly promoting HLA-E expression, we established gain-of- and loss-of-function models. We first compared HLA-E expression levels in U266 cells overexpressing CREB1 or CD56 (gain-of-function models) or U266 cells transduced with empty control vectors (CNT). We observed an increase in total HLA-E by western blot analysis (Fig. S3C) and in HLA-E surface expression in U266 cells overexpressing either CREB1 or CD56 (Fig. 2G, H). For loss-of-function studies, we used two approaches: genomic inhibition was obtained using shRNAs to silence CREB1 in OPM-2 cells, while pharmacological inhibition was achieved using the CREB1 inhibitor named 666-15 (CREBi). Both strategies led to reduced HLA-E protein expression in the whole cells, as shown by western blot analysis (Fig. S3D, E), and on the cell surface as measured by flow cytometry (Fig. 2I). The reduction of HLA-E surface expression was also confirmed in CD56-expressing patient-derived MM cells treated with the CREBi for 48 h (Fig. 2I). Overexpression of CREB1 induced HLA-E mRNA (Fig. 2J), while the opposite occurred with CREB1 silencing (Fig. 2J) or pharmacological inhibition of CREB1 (Fig. S3F). Similarly, we observed an increase of HLA-E mRNA levels by overexpression of CD56 and downregulation of HLA-E mRNA levels by CD56 silencing (Fig. 2K). Thus, we conclude that CREB1 modulates the expression of immune checkpoint HLA-E on MM cells, likely mediating immune escape.

### CREB1 regulates STAT1 signaling

**Fig. 3:**
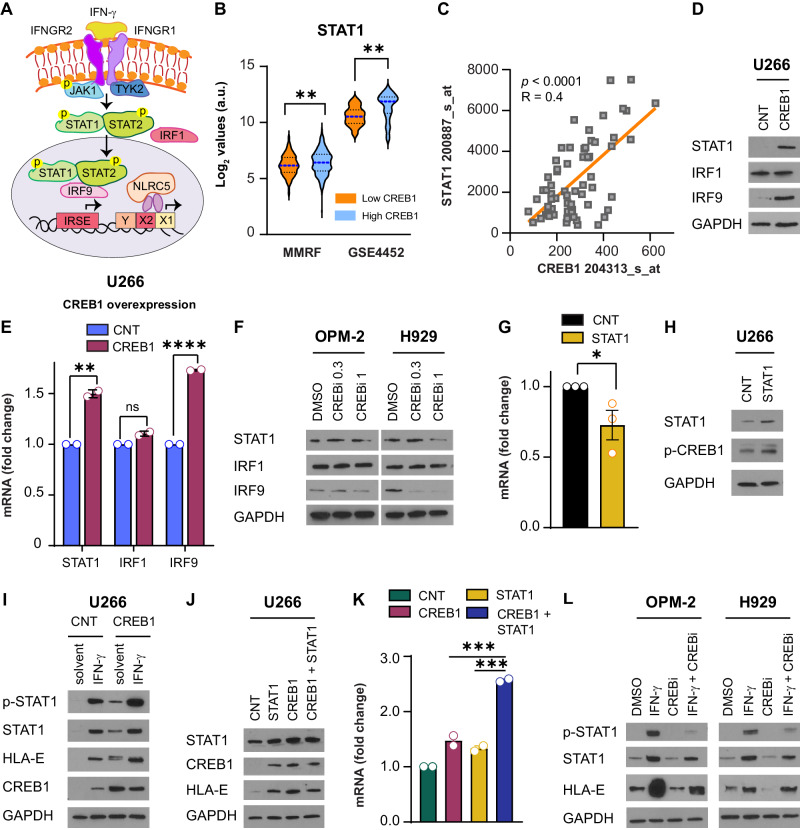
Both STAT1 and CREB1 regulate HLA-E expression. **A** Schema of IFN-γ signaling. IFN-γ signals via a receptor composed of two IFNGR chains (IFNGR1 and 2), which activate JAK1 and STAT1/STAT2. Phosphorylated STAT1/STAT2 complexes interact with IRF1 and IRF9, translocate to the nucleus, and bind to DNA on interferon sensitive response elements (ISRE). NLRC5 also binds to DNA to promote IFN-γ-related gene transcription. **B** STAT1 log_2_ expression values in patients with low or high CREB1 expression based on median cutoff of CREB1 expression. CoMMpass MMRF database: *n* = 809, *p* = 0.0015, ** and GSE4452 database: *n* = 65, *p* = 0.0013, **. Dashed blue lines indicate the median values; black dotted lines represent the 25^th^ and 75^th^ percentile. **C** Regression studies to correlate STAT1 (probe 200887_s_at) as dependent variable to CREB1 (probe 204313_at). *p* < 0.0001; *R* = 0.4. **D** Western blot analysis for STAT1, IRF1, IRF9, and GAPDH in U266 control cells (CNT) or U266 cells overexpressing CREB1. **E** STAT1, IRF1, and IRF9 mRNA fold change in U266 control cells (CNT) or U266 cells overexpressing CREB1. *n* = 2, t test, two-tailed; STAT1 *p* = 0.0031, **; IRF1 *p* = ns; IRF9 *p* < 0.0001, ****. **F** Western blot analysis for STAT1, IRF1, IRF9, and GAPDH in OPM-2 and H929 cells treated with DMSO, 666-15 (CREBi) 0.3 μM, and 666-15 (CREBi) 1 μM for 48 h. **G** CREB1 mRNA fold change in U266 control cells (CNT) or U266 cells overexpressing STAT1. *n* = 3, t test, two-tailed; *p* = 0.05, *. **H** Western blot analysis for STAT1, phospho-CREB1, and GAPDH in U266 control cells (CNT) or U266 cells overexpressing STAT1. **I** Western blot analysis for phospho-STAT1, STAT1, HLA-E, CREB1, and GAPDH in U266 control cells (CNT) or U266 cells overexpressing CREB1 treated with solvent or IFN-γ 1 ng/mL for 24 h. **J** Western blot analysis for STAT1, CREB1, HLA-E, and GAPDH in U266 control cells (CNT), U266 cells overexpressing STAT1, CREB1, or CREB1 + STAT1. **K** HLA-E mRNA fold change in the same conditions reported in panel **J**. *n* = 2, t test, two-tailed; CREB1 versus CREB1 + STAT1 *p* = 0.0004, ***; STAT1 versus CREB1 + STAT1 *p* = 0.0003, ***. **L** Western blot analysis for phospho-STAT1, STAT1, HLA-E, and GAPDH in OPM-2 and H929 cells treated with DMSO, IFN-γ 1 ng/mL, 666-15 (CREBi) 1 μM, and the combination of IFN-γ + CREBi for 24 h.

IFN-γ activates JAK-STAT1 and requires IRF family genes, such as IRF1, IRF2, IRF3, and IRF9 to promote gene transcription [18]; alternatively, NLR family CARD domain containing 5 (NLRC5) can form an MHC class I enhanceosome [19], binding to ATF1/CREB and the NFY-complex to activate gene transcription (Fig. 3A). The relationship between STAT1 and CREB1 pathways is largely unknown. We observed that STAT1, IRF1, IRF3, and IRF9 have variable expression in MM patient samples or MM cell lines, while NLRC5 is relatively low expressed (Fig. S4A, B). Patients with high CREB1 expression had statistically significant higher STAT1 levels compared with patients with low CREB1 expression (Fig. 3B). Linear regression analysis was also statistically significant between CREB1 and STAT1 as dependent, with a *p* value of <0.0001 and a R-squared coefficient of 0.4 (Fig. 3C). Conversely, the correlation between CREB1 and IRF9 was statistically significant (Fig. S5A) but not as robust (Fig. S5B), while there was no statistical correlation between CREB1 and IRF1 levels (Fig. S5A, C). To evaluate whether CREB1 was prompting STAT1 signaling, we overexpressed CREB1 in U266 cells. CREB1 increased the protein and mRNA levels of STAT1 and IRF9 but did not affect IRF1 levels, in agreement with the low correlation seen in patients (Fig. 3D, E). Treatment with CREBi (Fig. 3F) or shRNAs targeting CREB1 (Fig. S6A, B) reduced STAT1 and IRF9 levels but did not affect IRF1 expression. Interestingly, IFN-γ reduced mRNA CREB1 levels (Fig. S6C), and decreased total CREB1 and phospho-CREB1, the activated form (Fig. S6D). Similarly, STAT1 overexpression reduced mRNA CREB1 levels with no effects on protein levels (Fig. 3G, H). This indicates that STAT1 and CREB1 can cooperate in inducing HLA-E, but STAT1 negatively regulates CREB1, suggesting a potential feedback loop. We then overexpressed CREB1 in U266 cells and either added IFN-γ to the culture or concomitantly overexpressed STAT1. We observed that IFN-γ treatment (Fig. 3I) or the combined overexpression of STAT1–CREB1 were additive in increasing the expression of HLA-E at the protein (Fig. 3J) and mRNA levels (Fig. 3K). In contrast, IFN-γ combined with CREB1 inhibition reduced HLA-E, STAT1, and phospho-STAT1 levels in CREB1 positive cell lines (Fig. 3L and Fig. S6E). IRF9 is downstream to STAT1; however, its overexpression did not affect HLA-E, CREB1, or STAT1 levels (Fig. S7A–C). Together, these data show that CREB1 also influences the expression of HLA-E indirectly by inducing STAT1 and IRF9 expression. In contrast, STAT1 limits CREB1 activation in a negative feedback manner.

### HLA-E expression is increased by immunomodulatory drugs and histone deacetylase inhibitors

**Fig. 4:**
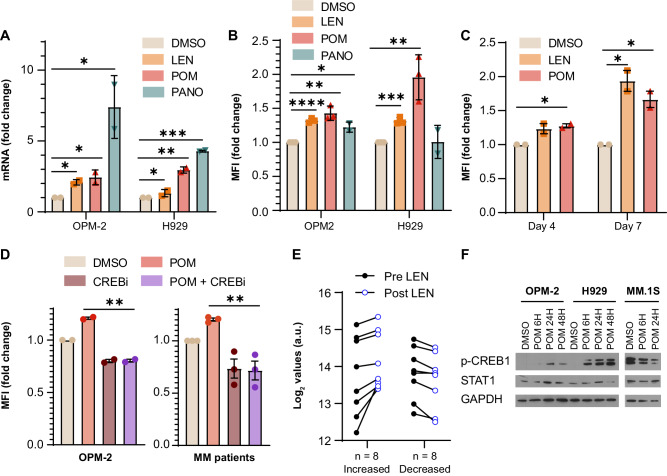
HLA-E expression is modulated by anti-MM drugs. **A** HLA-E mRNA fold change in OPM-2 and H929 cells treated with DMSO, lenalidomide (LEN) 1 μM for 72 h, pomalidomide (POM) 1 μM for 72 h, and panobinostat (PANO) 100 nM for 24 h. *n* = 2, t test, two-tailed. OPM-2: DMSO versus LEN *p* = 0.0149, *; DMSO versus POM *p* = 0.0293, *; DMSO versus PANO *p* = 0.05, *. H929: DMSO versus LEN *p* = 0.0349, *; DMSO versus POM *p* = 0.0063, **; DMSO versus PANO *p* = 0.0002, ***. **B** HLA-E MFI fold change in OPM-2 cells and H929 cells treated with DMSO, LEN 1 μM for 72 h, POM 1 μM for 72 h, and PANO 100 nM for 24 h. *n* = 3, t test, two-tailed. OPM-2: DMSO versus LEN *p* < 0.0001, ****; DMSO versus POM *p* = 0.0021, **; DMSO versus PANO *p* = 0.0105, *. H929: DMSO versus LEN *p* = 0.0004, ***; DMSO versus POM *p* = 0.0075, **; PANO *p* = 0.97, ns. **C** HLA-E MFI fold change in OPM-2 cells treated with DMSO, LEN 0.1 μM, or POM 0.1 μM for 4 and 7 days. *n* = 2, t test, two-tailed. Day 4: DMSO versus LEN *p* = 0.05, *; DMSO versus POM *p* = 0.0115, *; day 7: DMSO versus LEN *p* = 0.0255, *; DMSO versus POM *p* = 0.0313, *. **D** HLA-E MFI fold change in OPM-2 cells and MM patient samples treated with DMSO, POM 1 μM, 666-15 (CREBi) 0.3 μM, or POM + CREBi. OPM-2: OPM-2 cells were treated for 72 h. *n* = 2, t test, two-tailed; POM versus POM + CREBi *p* = 0.0018, **. MM patients: patient samples were treated for 48 h. *n* = 3, t test, two-tailed; POM versus POM + CREBi *p* = 0.0059, **. **E** Gene-expression profiling for HLA-E in *n* = 16 relapsed refractory patients with MM with matched pre- and post-lenalidomide (LEN) data from dataset GSE8546. **F** Western blot analysis for p-CREB1, STAT1, and GAPDH in OPM-2, H929, and MM.1S cells treated with DMSO and POM 1 μM for 6, 24, and 48 h.

Several medications used to treat patients with MM, including IMiDs (lenalidomide-LEN and pomalidomide-POM), and histone deacetylase (HDAC) inhibitor, panobinostat (PANO), activate the phosphorylation of STAT1, resulting in the transcription of IFN-γ target genes [20–22]. Since HLA-E is also an IFN-γ-stimulated gene, we evaluated HLA-E expression with treatment with 1 μM LEN, 1 μM POM, and 100 nM PANO for 24–72 h. At variable degrees, all three drugs increased the expression of HLA-E both at the mRNA (Fig. 4A) and protein levels (Fig. 4B and Fig. S8A). Interestingly, HLA-E expression kept increasing over time in cells treated with LEN or POM for longer time points (4 or 7 days), even using a lower concentration of 0.1 μM (Fig. 4C and Fig. S8B). This suggests that HLA-E regulation by IMiDs is a persistent phenomenon. In contrast, proteasome inhibitors (PIs), such as bortezomib (BTZ) and carfilzomib (CFZ) decreased the expression of HLA-E either as single agents or in combination with LEN and POM (Fig. S8C), adding a new rationale to the combination of IMiDs and PIs in patients.

Co-incubation of IMiDs and CREBi also reduced HLA-E levels in both cell lines and patient samples (Fig. 4D and Fig. S8D). We then evaluated a dataset of patients with gene-expression profiling data pre- and post-lenalidomide treatment, with half of the patients increasing HLA-E expression with lenalidomide and half of them having stable or decreased levels (Fig. 4E). Interestingly, this subgrouping correlated with CREB1 expression but not STAT1 expression (Fig. S8E). We then observed that LEN and POM had a time-dependent effect on the phosphorylation of CREB1 which varied between cell lines (Fig. 4F and Fig. S8F), potentially explaining the variability of HLA-E modulation in the patient samples.

### CD56 expression in patients changes the NK cell immune repertoire

**Fig. 5:**
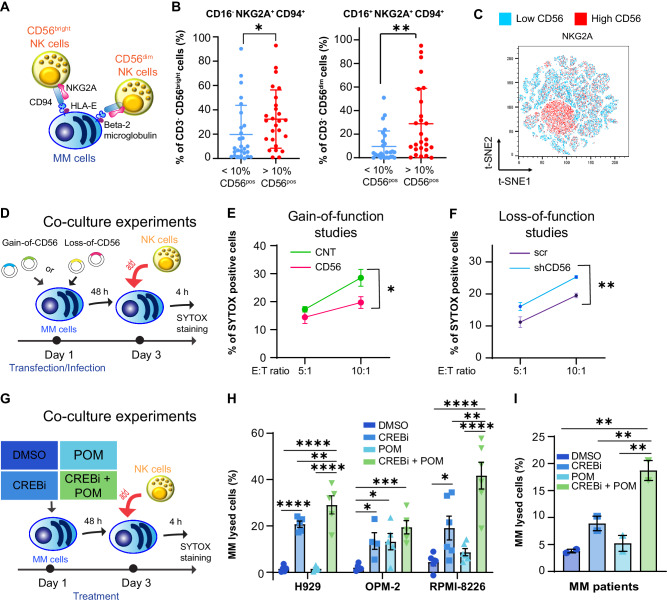
NK cell-mediated cytotoxicity is increased by CREB1 inhibition. **A** Cellular interactions between HLA-E^+^ MM cells and CD94^+^ NKG2A^+^ NK cells (either CD56^bright^ or CD56^dim^). **B** Percentages of CD3^−^ CD56^bright^ CD16^−^ CD94^+^ NKG2A^+^ NK cells (t test, two-tailed; *p* = 0.021, *) or CD3^−^ CD56^dim^ CD16^+^ CD94^+^ NKG2A^+^ NK cells (t test, two-tailed; *p* = 0.004, **) in patients with <10% of CD56-expressing clonal MM cells (*n* = 27) or >10% of CD56-expressing clonal MM cells (*n* = 26). **C** t-SNE analysis combining CD94^+^ NKG2A^+^ cells in the two conditions. **D** Schema of co-culture experiments using gain-of- or loss-of-function cells. Freshly isolated NK cells were co-cultured with MM cells for 4 h. SYTOX staining was used to distinguish viable from dead MM cells. **E** U266 control cells (CNT) or U266 cells overexpressing CD56 (CD56) were incubated with NK cells derived from three healthy donors (E:T ratio 5:1 *p* = 0.11, ns; E:T ratio 1:10 *p* = 0.014, *). **F** H929 scrambled cells (scr) or H929 cells silenced for CD56 (shCD56) were incubated with NK cells derived from two healthy donors (E:T ratio 5:1 *p* = 0.08, ns; E:T ratio 10:1, *p* = 0.009, **). **G** Schema of co-culture experiments in cells pretreated for 48 h with DMSO, 666-15 (CREBi) 0.3 μM, pomalidomide (POM) 1 μM, or combination of CREBi with POM (CREBi + POM). Freshly isolated NK cells were co-cultured with MM cells for 4 h. SYTOX staining was used to distinguish viable from dead MM cells. **H** NK cell-mediated cytotoxicity by SYTOX staining in H929, OPM-2, and RPMI-8226 cells treated with DMSO, CREBi 0.3 μM, POM 1 μM, or combination of CREBi with POM (CREBi + POM) with or without NK cells. NK cells were isolated from *n* = 6 different healthy donors. E:T ratio was 5:1. % of lysis is calculated as described in the “method” section. H929: ANOVA *p* < 0.0001; “DMSO + NK cells” versus “CREBi + NK cells” *p* < 0.0001, ****; “DMSO + NK cells” versus “CREBi + POM + NK cells” *p* < 0.0001, ****; “CREBi + NK cells” versus “CREBi + POM + NK cells” *p* = 0.0041, **; “POM + NK cells” versus “CREBi + POM + NK cells” *p* < 0.0001, ****. OPM-2: ANOVA *p* = 0.0041; “DMSO + NK cells” versus “CREBi + NK cells” *p* = 0.0149, *; “DMSO + NK cells” versus “POM + NK cells” *p* = 0.0124, *; “DMSO + NK cells” versus “CREBi + POM + NK cells” *p* = 0.0005, ***; RPMI-8226: ANOVA *p* < 0.0001; “DMSO + NK cells” versus “CREBi + NK cells” *p* = 0.0261, *; “DMSO + NK cells” versus “CREBi + POM + NK cells” *p* < 0.0001, ****; “CREBi + NK cells” versus “CREBi + POM + NK cells” *p* = 0.001, **; “POM + NK cells” CREBi + POM “combo + NK cells” *p* < 0.0001, ****. **I** NK cell-mediated cytotoxicity by SYTOX staining in MM patient samples treated with DMSO, CREBi 0.3 μM, POM 1 μM, or combination of CREBi with POM (CREBi + POM) with or without NK cells. NK cells were isolated from *n* = 2 different healthy donors. ANOVA *p* = 0.0050; “DMSO + NK cells” versus “CREBi + POM + NK cells” *p* = 0.0015, **; “CREBi + NK cells” versus “CREBi + POM + NK cells” *p* = 0.0071, **; “POM + NK cells” versus “CREBi + POM + NK cells” *p* = 0.0022, **.

NK cells develop from common lymphoid progenitors into CD56^bright^ NKG2A^+^ CD16^−^ to then mature into CD56^dim^ NKG2A^+/−^ CD16^+^ NK cells, with NKG2A being the inhibitory receptor that recognizes HLA-E (Fig. 5A and as shown in Freud et al. [2, 23]). The education and plasticity of NK cells depend on their interactions with the surrounding cells [24]. Therefore, we hypothesize that MM cells could influence the immune phenotype of NK cells. We evaluated different NK cellular subsets and we identified greater percentages of inhibitory NK cells (CD3^−^ CD56^bright^ CD16^−^ NKG2A^+^ CD94^+^ NK cells: Low CD56 group: median = 8.54%, range: 0–90.2%; High CD56 group: median = 31%, range: 0.93–92.9%, Mann–Whitney *p* = 0.0211, * and CD3^−^ CD56^dim^ CD16^+^ NKG2A^+^ CD94^+^ NK cells: Low CD56 group: median = 3.9%, range: 0–50.9%, High CD56 group: median = 14.4%, range: 0–95%, Mann–Whitney *p* = 0.0040, **) in patients with more than 10% of CD56-expressing clonal MM cells (Fig. 5B, C). Since HLA-E expression is proportional to CD56 expression and influences NK cell phenotypes, our data suggest that greater levels of HLA-E on the MM cells can drive NK cells toward a more immunosuppressive phenotype, with reduced cytotoxicity abilities.

### CD56 and CREB1 influence NK cell-mediated cytotoxicity toward MM cells

To evaluate if CREB1 signaling could modulate NK cell-mediated cytotoxicity, we then performed co-culture experiments in different conditions. First, cells either overexpressing (U266 cells) or silenced for CD56 (H929 cells) with their relative controls were co-incubated for 4 h with NK cells at Effector:Target (E:T) ratio of 5:1 and 10:1 (Fig. 5D). We observed reduced NK cell-mediated cytotoxicity in U266 overexpressing CD56 (Fig. 5E) and increased NK cell-mediated cytotoxicity in H929 silenced for CD56 (Fig. 5F). We then pretreated MM cell lines or MM patients’ samples with DMSO, 666-15 (CREBi), POM, or combination of both (POM + CREBi) for 48 h, followed by the addition of freshly isolated NK cells for another 4 h (Fig. 5G). CREBi was synergistic with LEN [16] and POM (Fig. S9A) in the absence of NK cells. However, the specific combination of CREBi and POM increased the killing ability of allogeneic healthy donor-derived NK cells more than the single drugs (Fig. 5H, I), without inducing cell death of NK cells (Fig. S9B). This suggests that CREB1 inhibition can restore NK cell-mediated cytotoxicity against MM cells.

## Discussion

To prevent MM disease progression, therapeutic drugs must target not only the MM cells but also the surrounding TME. Advancing the response to cancer immunotherapies in MM is strongly linked to the ability to counteract immune escape. Several studies have highlighted the presence of a perturbed T and NK cell repertoire expressing exhausted markers such as LAG3, TIGIT, and TIM3 either at diagnosis or after therapies in patients with MM [4, 25–27]. Fewer studies have focused on anti-inflammatory or checkpoint inhibitory markers on MM cells. While PD-1 blockade has not been successful in MM, with clinical trials utilizing PD-1 inhibitors being suspended prematurely for increased mortality [28, 29], CD94/NKG2A-HLA-E axis represents an important immune checkpoint to study. Targeting NKG2A by monoclonal antibodies [8] or protein expression blockers [30] has been validated in preclinical models but showed variable activity in clinical trials [31, 32]. Our work illuminates a connection between CD56/CREB1 signaling and immune escape. While it was known that IFN-γ via STAT1 could increase HLA-E levels, we here indisputably prove that CREB1 and STAT1 both contribute to HLA-E expression in myeloma, with CREB1 binding to HLA-E promoter and CREB1 inhibitors decreasing HLA-E expression in cell lines and patient samples. JAK1 inhibitors, such as ruxolitinib, or STAT1 inhibitors [33] can potentially be used to block HLA-E expression. While ruxolitinib is generally well tolerated and has minimal anti-MM activity [34], STAT inhibitors usually block different STATs, leading to toxicities but also compensatory mechanisms [35]. Therefore our work paves the way to an alternative strategy based on the inhibition of CREB1, which can directly induce MM cell apoptosis [16] but also modulate the immune system. The expression of HLA-E and other IFN-γ related genes in MM highlights the presence of an immune phenotype in a proportion of patients with MM, which is further triggered and maintained by immunomodulatory drugs; CREB1 inhibition was able to exploit this phenotype potentiating the effect of pomalidomide in the presence of NK cells, without affecting NK cell viability.

Compared to normal plasma cells, HLA-E expression is upregulated in the precursor forms MGUS and SMM and in overt MM, potentially inducing immune escape of malignant clones by the increase of NKG2A^+^CD94^+^ NK cells. Other groups identified HLA-E in circulating tumor cells derived from pancreatic ductal adenocarcinoma [36]. We discovered that both CD56 and HLA-E can be detected at variable levels in extracellular vesicles (EVs) derived from MM cell lines (Fig. S10A–C). Overexpression of CREB1 and CD56 in U266 cells increased the presence of HLA-E mRNA and protein in EVs (Fig. S10D–F), while treatment with CREBi reduced HLA-E levels (Fig. S10G, H). Targeting HLA-E expression could hence be a strategy to prevent MM progression or to reduce bloodstream dissemination, by promoting enhanced immune surveillance and removal of decoy particles in the circulation. Detection of circulating HLA-E positive EVs might also be a potential marker of early stages of MM.

Alterations in NK cell phenotype and function occur in patients with MM [4–6]. Herein, we observed that the presence of active CD56/CREB1 signaling in MM cells is associated with an increase of inhibitory NKG2A^+^CD94^+^ NK cells, similarly to the phenotype observed in patients with t(4;14) [11]. It is unclear whether this is a mere association, or it is the result of the modeling of the immune system by the MM cells themselves [37]. Nevertheless, a better understanding of how MM cells shape the immune system is crucial to improve response to immunotherapies in MM, by reverting immunosuppressive phenotypes. Finally, while this report focuses only on NK cells, other players are involved in immune escape, including NKG2A^+^CD94^+^ T cells, myeloid cells, and cancer associated fibroblasts [38]. Given CREB1’s broad role in immunity [39], the interactions with other immune cell populations remain vastly unexplored and will be addressed in future studies.

In conclusion, our study defines the role of CREB1 in modulating HLA-E expression; CREB1 inhibition improves NK cell-mediated cytotoxicity in MM and represents a novel strategy to tackle immune escape.

### Supplementary information

Supplementary materials

## Data Availability

This study did not generate new unique reagents. Cell lines can be shared upon reasonable request from the lead contact without restriction. Further information and requests for resources and reagents should be directed to and will be fulfilled by the lead contact, FC (francesca.cottini@osumc.edu).
