# Supplementary material for: CREB1 promotes expression of immune checkpoint HLA-E leading to immune escape in multiple myeloma
Source: Leukemia. 2024 Jun 20;38(8):1777–86. doi: 10.1038/s41375-024-02303-w (PMC11286514; doi:10.1038/s41375-024-02303-w)
Supplement: Supplementary file 1 — Supplementary materials [file 41375_2024_2303_MOESM1_ESM.pdf]

Title: CREB1 promotes expression of immune checkpoint HLA-E leading to immune escape in Multiple Myeloma

Authors:

Aya Ismael[1], Allen J. Robinette[1], Laila Huric[1], Jamie Schuetz[2], Kameron Dona[1], Don Benson[1], Emanuele Cocucci[3], and Francesca Cottini[1]*

[1] The Ohio State University College of Medicine, Department of Internal Medicine, Division of Hematology, Columbus, OH, USA.

[2] The Ohio State University College of Veterinary Medicine, Comparative Pathology and Digital Imaging Shared Resource Main Laboratory, Columbus, OH, USA.

[3] The Ohio State University College of Pharmacy, Department of Pharmaceutics and Pharmacology, Columbus, OH, USA.

***Correspondence:**

Francesca Cottini, MD (lead contact)

Assistant Professor of Medicine

Division of Hematology

The Ohio State University, College of Medicine

385G Wiseman Hall | 400 W 12th Ave |

Columbus OH 43210-1240 USA

Email: Francesca.cottini@osumc.edu

Supplementary Contents

Supplementary Methods

Reagents, Real-Time quantitative PCR primers, antibodies for western blot, Chromatin Immunoprecipitation (ChIP) protocol, ChIP-qPCR, and ChIP-sequencing, RNA sequencing experiments, plasmid vectors, Immunohistochemistry, Extracellular vesicles (EV) isolation, Viability and cellular growth assays.

Supplementary Figures and Figure Legends

Figure S1: HLA-E expression in MM. Related to Figure 1.

Figure S2: In silico analysis for IFN-γ-related pathways. Related to Figure 2.

Figure S3: HLA-E modulation by CD56 or CREB1. Related to Figure 2.

Figure S4: STAT1 pathway in MM. Related to Figure 3.

Figure S5: *In silico* correlation of CREB1 with IRF9 or IRF1 in the GSE4452 dataset. Related to Figure 3.

Figure S6: CREB1 and STAT1 regulation. Related to Figure 3.

Figure S7: IRF9 overexpression in MM. Related to Figure 3.

Figure S8: Regulation of HLA-E expression by anti-MM drugs and CREB1 inhibitors. Related to Figure 4.

Figure S9: Cytotoxic effects of CREB1 inhibitors and/or IMiDs on myeloma cells and natural killer (NK) cells. Related to Figure 5.

Figure S10: HLA-E and its presence in extracellular vesicles (EVs). Related to discussion.

Supplementary Methods

Reagents

The following compounds and reagents were used: interferon-gamma (IFN-γ, R&D Systems, Cat. No. 285-IF, Lot# RAX2422021) in water; 666-15 (Tocris, Bio-Techne Corporation, Cat. No. 5661), lenalidomide (Millipore Sigma, Cat. No. SML2283), pomalidomide (Selleck Chemicals, Cat. No. S1567), panobinostat (MedChemExpress, Cat. No. HY-10224), bortezomib (Selleck Chemicals, Cat. No. S1013), and carfilzomib (Selleck Chemicals, Cat. No. S2853) in DMSO.

Real-Time quantitative PCR primers

The following primers were used: GAPDH_F: 5'- GAAGGTGAAGGTCGGAGTCA-3', GAPDH_R: 5'- GGGGTCATTGATGGCAACAATA-3', HLA-E_F: 5'-TTCCGAGTGAATCTGCGGAC-3', HLA-E_R: 5'-GTCGTAGGCGAACTGTTCATAC-3', CREB1_F: 5'-ATTCACAGGAGTCAGTGGATAGT-3', CREB1_R: 5'-CACCGTTACAGTGGTGATGG-3', IRF1_F: 5'-ATGCCCATCACTCGGATGC-3', IRF1_R: 5'-CCCTGCTTTGTATCGGCCTG-3', IRF9_F: 5'-GCCCTACAAGGTGTATCAGTTG-3', IRF9_R: 5'-TGCTGTCGCTTTGATGGTACT-3', STAT1_F: 5'-CAGCTTGACTCAAAATTCCTGGA-3', STAT1_R: 5'-TGAAGATTACGCTTGCTTTTCCT-3'.

Antibodies for western blot

The following primary antibodies were used: HLA-E (Abcam, Cat. No. ab2216, RRID:AB_302895), CD56 (Cell Signaling Technology, Cat. No. 3576, RRID:AB_2149540), CREB1 (Cell Signaling Technology, Cat. No. 9197, RRID:AB_331277), phospho-CREB1 (Ser 133) (Cell Signaling Technology, Cat. No. 9198, RRID:AB_2561044), GAPDH (Cell Signaling Technology, Cat. No. 3683, RRID:AB_1642205), CD63 (Invitrogen, Cat. No. 10628D, RRID:AB_2532983), phospho-STAT1 (Cell Signaling Technology, Cat. No. 7649, RRID:AB_10950970), STAT1 (Cell Signaling Technology, Cat. No. 14995, RRID:AB_2716280),

IRF1 (Cell Signaling Technology, Cat. No. 8478, RRID:AB_10949108), IRF3 (Cell Signaling Technology, Cat. No 11904, RRID:AB_2722521), IRF9 (Cell Signaling Technology, Cat. No. 76684, RRID:AB_2799885), NLRC5 (Santa Cruz Technology, sc-515668), alpha-actinin (Cell Signaling Technology, Cat. No. 3134, RRID:AB_2223798), and flotillin (BD Biosciences, Cat. No. 610821, RRID:AB_398140). The following secondary antibodies were used: anti-rabbit IgG HRP-linked (Cell Signaling Technology, Cat. No. 7074, RRID:AB_2099233) or anti-mouse IgG HRP-linked (Cell Signaling Technology, Cat. No. 7076, RRID:AB_330924).

Chromatin Immunoprecipitation (ChIP) protocol, ChIP-qPCR, and ChIP-sequencing

H929 cells were treated with DMSO or 666-15 at 2 μM for 24 hours. Protein-DNA complex chromatin extraction was performed using Magna ChIP™ A/G kit (Millipore, Burlington, MA, United States, Cat. No. 17-10085) starting from 5 million cells per condition. Immunoprecipitation was performed on 10 µg of crosslinked chromatin using anti-CREB1 antibody (Cell Signaling Technology, Cat. No. 9197, RRID:AB_331277) or isotype Ig, overnight at 4°C. The resulting DNA extract was assessed by real-time quantitative PCR to measure enrichment within the promoter region of HLA-E gene using the following specific primers (HLA-E_F promoter: 5'-TCCCGTTCCTCTCGTAACCTGGT-3'; HLA-E_R promoter: 5'-GTGGCGACGCTGATTGGCTTCT-3'; GAPDH_F promoter: 5'-CGACCACTTTGTCAAGCTCA-3' and GAPDH_R promoter: 5'-AGGGGTCTACATGGCAACTG-3'). Analysis was performed using the fold enrichment method. ChIP-Sequencing and post-processing of the raw data were performed by Active Motif, Inc (Carlsbad, CA, United States). Images were obtained using Integrative Genomics Viewer software version 2.16 (Broad Institute).

RNA sequencing experiments

RNA from MM cells in different conditions (3 independent experiments) was extracted using ReliaPrep™ RNA Miniprep Systems (Promega, Cat. No. Z6011). All RNA samples were evaluated for concentration by Qubit® and integrity using Agilent 2100 Bioanalyzer, or the Perkin

Elmer LabGX. Standard RNA-Seq (60-80M read pairs) was performed using Illumina NovaSeq 6000 SP PE 150bp format. Service and post-processing of the raw data were performed by the IGM Genomic Services Lab of The Research Institute at Nationwide Children's Hospital, Columbus, Ohio.

Plasmid vectors

For gain-of-function studies, the following plasmids were used: CD56 plasmid (MHS6278-202802080; clone ID number 5590188, Dharmacon, a Horizon Discovery group company, Lafayette, CO, United States); CREB1 wild-type plasmid (PT3457-5, Cat. No. 631925, Clontech Laboratories, Inc, a Takara Bio Company, Kusatsu, Shiga, Japan); eGFP STAT1 wild-type plasmid cDNA (Addgene plasmid # 12301; http://n2t.net/addgene:12301; RRID:Addgene_12301); pCMV3 empty vector, called CNT in the manuscript (Cat. No. CV011, Sino Biological US, Wayne, PA, United States); and IRF9 wild-type plasmid (HG12602-UT, Sino Biological US). For loss-of-function studies, pLKO.1-TRC control was used as control vector, called scr in the manuscript (Addgene plasmid # 10879, RRID: Addgene_10879), while shRNAs against CD56 (RHS3979-201791074, TRCN0000073460) and CREB1 (RHS3979-201739582, TRCN0000007309) were obtained from Dharmacon.

Immunohistochemistry

Samples used for immunohistochemistry were obtained from the institutional database after approval from the IRB Committee was granted for the study (OSU-23131). 4 µm sections were cut from formalin-fixed paraffin-embedded (FFPE) tissue samples using the Leica Bond III system. The tissue was pretreated for 20 minutes with BOND Epitope Retrieval Solution 2, an EDTA based pH 9.0 solution (Leica Biosystems, Cat. No. AR9640). Sections were then washed in tris-buffered saline (TBS, pH 7.4, BIO-RAD) and the antibody was incubated for 15 minutes at a 1:1000 dilution. Tissue sections were visualized using the Leica BOND Polymer Refine Detection solution (Leica Biosystems, Cat. No. DS9800) which uses a biotin-free, polymer HRP

DAB detection system. Tissue sections were then counterstained with hematoxylin. Optimization was performed using different antibody dilutions (1:100, 1:250, 1:500, and 1:1000) and low or high PH retrieval conditions. Images were acquired with an Axiocam 208 camera (3840 x 2160 pixels) mounted on a ZEISS Axioscope 5 equipped with plan lenses (5x/0.12; 10x/0.25; 20x/0.40; 40x/0.65; 100x/1.25) using ZEN Microscopy Software (Zeiss). The following primary antibody was used: anti-HLA-E (Abcam, Cat. No. ab2216, RRID:AB_302895).

Extracellular vesicles (EV) isolation

MM cells were first cultured in Opti-MEM™ I Reduced Serum Medium (Gibco) for 24 hours and then either modified by plasmid transfection or treated with the CREB1 inhibitor, 666-15. Supernatants were collected following a standard differential centrifugation protocol. Briefly, supernatants were first centrifuged at 2,000 × g for 5 min at 4°C to remove dead cells and cell debris; supernatants were then collected and centrifuged at 100,000 g for 60 min at 4°C (Optima-Max-XP ultracentrifuge, Beckman Coulter) to pellet EVs. RNA was extracted using ReliaPrep™ RNA Miniprep Systems; lysates were obtained by incubation in sample buffer before SDS-electrophoresis.

Long-term treatment with lenalidomide and pomalidomide

OPM-2 cells were plated in RPMI-supplemented medium containing DMSO at 0.1 μM, lenalidomide (LEN) at 0.1 μM and pomalidomide (POM) at 0.1 μM to assess HLA-E changes by flow cytometry and real-time PCR at days 4 and 7 of treatment. We used lower doses of LEN or POM compared to the experiments in Figures 4A-B to avoid excessive cell death. Every 48 hours, the medium of the three conditions was replaced with fresh medium, containing either DMSO, LEN or POM at the same 0.1 μM concentration. Flow staining was performed at days 4 and 7, while RNA extraction for real-time PCR was performed at day 7.

Viability and cellular growth assays

Viability of MM cells was evaluated by Thiazolyl Blue tetrazolium bromide, MTT (Sigma-Aldrich, St. Louis, MO, United States) colorimetric survival assay. MM cells (10,000-15,000) were plated in RPMI-1640 supplemented medium. At the various time points (48 or 72 hours), 10 μl of 5 mg/mL MTT was added to the cells. After 4 hours of incubation at 37 °C, the medium was discarded, and MTT stop solution (2-propanol, Millipore Sigma with 1 N Hydrochloric acid, Millipore Sigma) was used to dissolve the MTT metabolic products. Absorbance was read at 570 nm, and the background was subtracted at 630 nm, using a Biotek Cytation 5 Cell Imaging Multi-Mode Reader equipped with Gen5 v3.10 software (Agilent). Synergy was calculated by Chou-Talalay method using CompuSyn software.

Supplementary Figure Legends

Figure S1. HLA-E expression in MM. Related to Figure 1.

(A) Flow cytometry staining for HLA-E in patients with Multiple Myeloma (MM).

(B) Mean fluorescence intensity (MFI) levels of HLA-E in n = 3 patients with MM and in a panel of MM cell lines.

(C) Immunohistochemistry staining for HLA-E (20X) in newly diagnosed patients with MM (n = 4).

(D) HLA-E mRNA fold change in MM.1S and MM.1R cells treated with solvent or IFN-γ 1 ng/mL for 24 hours. n = 2, t test, two-tailed; MM.1S *p* = 0.0080, **; MM.1R *p* = 0.0203, *.

(E) Western blot analysis for HLA-E and GAPDH in MM.1S and MM.1R treated with solvent or IFN-γ 1 ng/mL for 24 hours.

Figure S2. In silico analysis for IFN-γ-related pathways. Related to Figure 2.

(A-B) Enrichment plots of IFN-driven pathways from C2_CP_REACTOME_INTERFERON_SIGNALING and C2_CP_PID_IFNG_PATHWAY in the

MMRF CoMMpass dataset (**A**) or GSE4452 dataset (**B**). Patients are divided based on median cutoff of CREB1 expression. NES, normalized enrichment score. FDR, false discovery rate.

(C) Enrichment plot of C2_CP_REACTOME INTERFERON_GAMMA_SIGNALING gene set in U266 control cells (CNT) or U266 overexpressing CD56. n = 3 replicates.

(D) Heat map with the genes of C2_CP_REACTOME INTERFERON_GAMMA_SIGNALING in U266 control cells (CNT) or U266 overexpressing CD56. n = 3 replicates.

(E) Heat map with the genes of C2_CP_REACTOME INTERFERON_GAMMA_SIGNALING in U266 control cells (CNT) or U266 overexpressing CREB1. n = 2 replicates.

Figure S3. HLA-E modulation by CD56 or CREB1. Related to Figure 2.

(A) Analysis of CREB1 binding in the human B-cell line GM12878 by ChIP-seq from the ENCODE Project. Shown is the mapping track of sequence reads at the HLA-E locus on chromosome 6.

(B) Fold enrichment for HLA-E promoter binding in H929 cells treated with DMSO or 666-15 (CREBi) 2 μM for 24 hours.

(C) Western blot analysis for HLA-E in U266 control cells (CNT) or cells overexpressing CREB1 (Left) or in U266 control cells (CNT) or cells overexpressing CD56 (Right) at 48 hours after transfection. GAPDH is used as normalizer; CD56 and CREB1 are shown to confirm overexpression.

(D) Western blot analysis for HLA-E, CREB1, and GAPDH in OPM-2 scrambled cells (scr) or cells silenced for CREB1 (shCREB1).

(E) Western blot analysis for HLA-E and actinin in OPM-2 and H929 cells treated with DMSO, 666-15 (CREBi) 0.3 μM, and CREBi 1 μM for 72 hours.

(F) HLA-E mRNA fold change in OPM-2 and H929 cells treated with DMSO and 666-15 (CREBi) 0.3 μM for 24 hours. n = 3, t test, two-tailed; OPM-2 $p = 0.0074$, **; and H929 $p = 0.0001$, ***.

Figure S4. STAT1 pathway in MM. Related to Figure 3.

(A) NLRC5, IRF1, IRF3, IRF9, and STAT1 $\log_2$ expression values in n = 809 patients with MM from the MMRF CoMMpass dataset. Dashed lines indicate the median values; dotted lines represent the 25th percentile and the 75th percentile.

(B) Western blot analysis for NLRC5, IRF1, IRF3, IRF9, and STAT1 in a panel of MM cell lines. The same lysates were run in duplicates. GAPDH was re-blotted on each membrane.

Figure S5. *In silico* correlation of CREB1 with IRF9 or IRF1 in the GSE4452 dataset. Related to Figure 3.

(A) IRF9 and IRF1 $\log_2$ mRNA values in patients with low or high CREB1 expression based on median cutoff of CREB1 expression. IRF9 $p = 0.0012$, **; IRF1 p = not significant, ns. Dashed blue lines indicate median values; dotted lines represent the 25th and 75th percentile.

(B) Regression studies to correlate IRF9 (probe 203882_at) as dependent variable to CREB1 (probe 204313_at). $p < 0.0001$; R = 0.2533.

(C) Regression studies to correlate IRF1 (probe 202531_at) as dependent variable to CREB1 (probe 204313_at). $p = 0.1714$, ns; R = 0.029.

Figure S6. CREB1 and STAT1 regulation. Related to Figure 3.

(A) STAT1, IRF1, and IRF9 mRNA fold change in OPM-2 scrambled cells (scr) or OPM-2 cells silenced for CREB1 (shCREB1). n = 3, t test, two-tailed; STAT1 $p = 0.038$, *; IRF1 $p = 0.32$, ns; IRF9 $p = 0.017$, *.

(B) Western blot analysis for STAT1, IRF9, and GAPDH of OPM-2 scrambled cells (scr) and OPM-2 cells silenced for CREB1 (shCREB1). Optical densities (OD) ratio for IRF9 normalized to GAPDH is reported in the figure.

(C) CREB1 mRNA fold change in OPM-2, H929, RPMI-8226, U266, MM.1S, and MM.1R treated with solvent or IFN-γ 1 ng/mL for 24 hours. n = 2, t test, two-tailed; *p* values are 0.0002, ***; 0.05, *; 0.00002, ****; 0.03, *; 0.003, **; and 0.027, * in the order presented.

(D) Western blot analysis for phospho-CREB1, total CREB1, and GAPDH in OPM-2, H929, RPMI-8226, and U266 cells treated with solvent or IFN-γ 1 ng/mL for 24 hours.

(E) HLA-E MFI fold change in OPM-2 and H929 treated with DMSO, IFN-γ 1 ng/mL, 666-15 (CREBi) 1 μM, or the combination of IFN-γ + CREBi. n = 2, t test, two tailed; OPM-2 cells: IFN-γ versus CREBi *p* = 0.0002, ***; IFN-γ versus IFN-γ + CREBi *p* = 0.0004, ***. H929 cells: IFN-γ versus CREBi *p* < 0.0001, ****; IFN-γ versus IFN-γ + CREBi *p* = 0.0002, ***.

Figure S7. IRF9 overexpression in MM. Related to Figure 3.

(A) IRF9 mRNA fold change in U266 control cells (CNT) or U266 cells overexpressing IRF9.

(B) HLA-E MFI fold change in U266 control cells (CNT) or U266 cells overexpressing IRF9. n = 2, t test, two-tailed; *p* = 0.59, ns.

(C) HLA-E, CREB1, and STAT1 mRNA fold change in U266 control cells (CNT) or U266 cells overexpressing IRF9. n = 2, t test, two-tailed; HLA-E *p* = 0.16, ns; CREB1 *p* = 0.3, ns; STAT1 *p* = 0.90, ns.

Figure S8. Regulation of HLA-E expression by anti-MM drugs and CREB1 inhibitors. Related to Figure 4.

(A) Western blot analysis for HLA-E and GAPDH in H929 cells treated with DMSO, LEN 1 μM, LEN 3 μM, POM 0.5 μM, or POM 1 μM for 72 hours; OPM-2 and H929 cells treated with DMSO and PANO 100 nM for 24 hours.

(B) HLA-E mRNA fold change in OPM-2 cells treated with DMSO, LEN 0.1 μM or POM 1 μM for 7 days. n = 2, t test, two-tailed. DMSO versus LEN *p* = 0.0024, **; DMSO versus POM *p* = 0.0098, **.

(C) Western blot analysis for HLA-E and GAPDH in OPM-2 cells treated with DMSO, bortezomib (BTZ) 5 nM, pomalidomide (POM) 1 μM, lenalidomide (LEN) 1 μM, or carfilzomib (CFZ) 5 nM either as single agents or in combination for 24 hours.

(D) Western blot analysis for HLA-E and GAPDH in H929 cells treated with DMSO, pomalidomide (POM) 1 μM, 666-15 (CREBi) 0.3 μM, or combination of POM and CREBi (POM + CREBi) for 72 hours.

(E) CREB1 (probe 877_at) and STAT1 (probe 32859_at) log_2 mRNA expression values in n = 8 with HLA-E increased post-lenalidomide (up) or decreased post-lenalidomide (down). CREB1 $p = 0.05$, *; STAT1 $p = 0.27$, ns. Solid black lines indicate the median values; dotted black lines represent the 25th and 75th percentiles.

(F) Western blot analysis for phospho-CREB1, STAT1, and GAPDH in OPM-2, H929, and MM.1S cells treated with DMSO or lenalidomide (LEN) 1 μM for 6, 24, and 48 hours.

Figure S9. Cytotoxic effects of CREB1 inhibitors and/or IMiDs on myeloma cells and natural killer (NK) cells. Related to Figure 5.

(A) Viability analysis by MTT assay in H929 and OPM-2 cells treated with DMSO, pomalidomide (POM) 1 μM and 666-15 (CREBi) at 0.1-3 μM for 72 hours. n = 2, t test, two-tailed. OPM-2: POM versus DMSO $p = 0.018$, *; POM versus CREBi 0.1 μM $p = 0.009$, **; POM versus CREBi 0.3 μM $p = 0.014$, *; POM versus CREBi 0.5 μM $p = 0.012$, *; POM versus CREBi 1 μM $p = 0.004$, **. H929: POM versus DMSO $p < 0.0001$, ****; POM versus CREBi 0.1 μM $p = 0.019$, *; POM versus CREBi 0.3 μM $p = 0.024$, *; POM versus CREBi 0.5 μM $p = 0.05$, *; POM versus CREBi 1 μM $p = 0.029$, *. Inserts show heat map of the synergic effects calculated by Chou-Talalay. CI stays for combination index.

(B) Cell death analysis by SYTOX staining of NK cells isolated from 3 healthy donors and treated for 48 hours with DMSO, 666-15 (CREBi) 0.3 μM, or CREBi 1 μM.

Figure S10. HLA-E and its presence in extracellular vesicles (EVs). Related to Discussion.

(A-C) EVs isolated from OPM-2, KMS-11, and H929 plated at different cellular concentrations in Opti-MEM medium. Western blot and Ponceau S staining are shown.

(D) HLA-E mRNA fold change in extracellular vesicles (EVs) isolated from U266 control cells (CNT) or U266 cells overexpressing CREB1. n = 2, t test, two-tailed; $p = 0.05$, *.

(E) HLA-E mRNA fold change in EVs isolated from U266 control cells (CNT) or U266 cells overexpressing CD56. n = 2, t test, two-tailed; $p = 0.047$, *.

(F) Western blot analysis for HLA-E, GAPDH, and CD63 using protein content isolated from EVs derived from U266 control cells (CNT) or U266 cells overexpressing CD56.

(G) Western blot analysis for HLA-E and flotillin using protein content isolated from EVs derived from OPM-2 cells treated with DMSO or 666-15 (CREBi) 0.3 μM for 48 hours.

(H) HLA-E mRNA fold change in EVs isolated from OPM-2 cells treated with DMSO or 666-15 (CREBi) 0.3 μM for 48 hours. n = 2, t test, two-tailed; $p = 0.0177$, *.

Figure S1

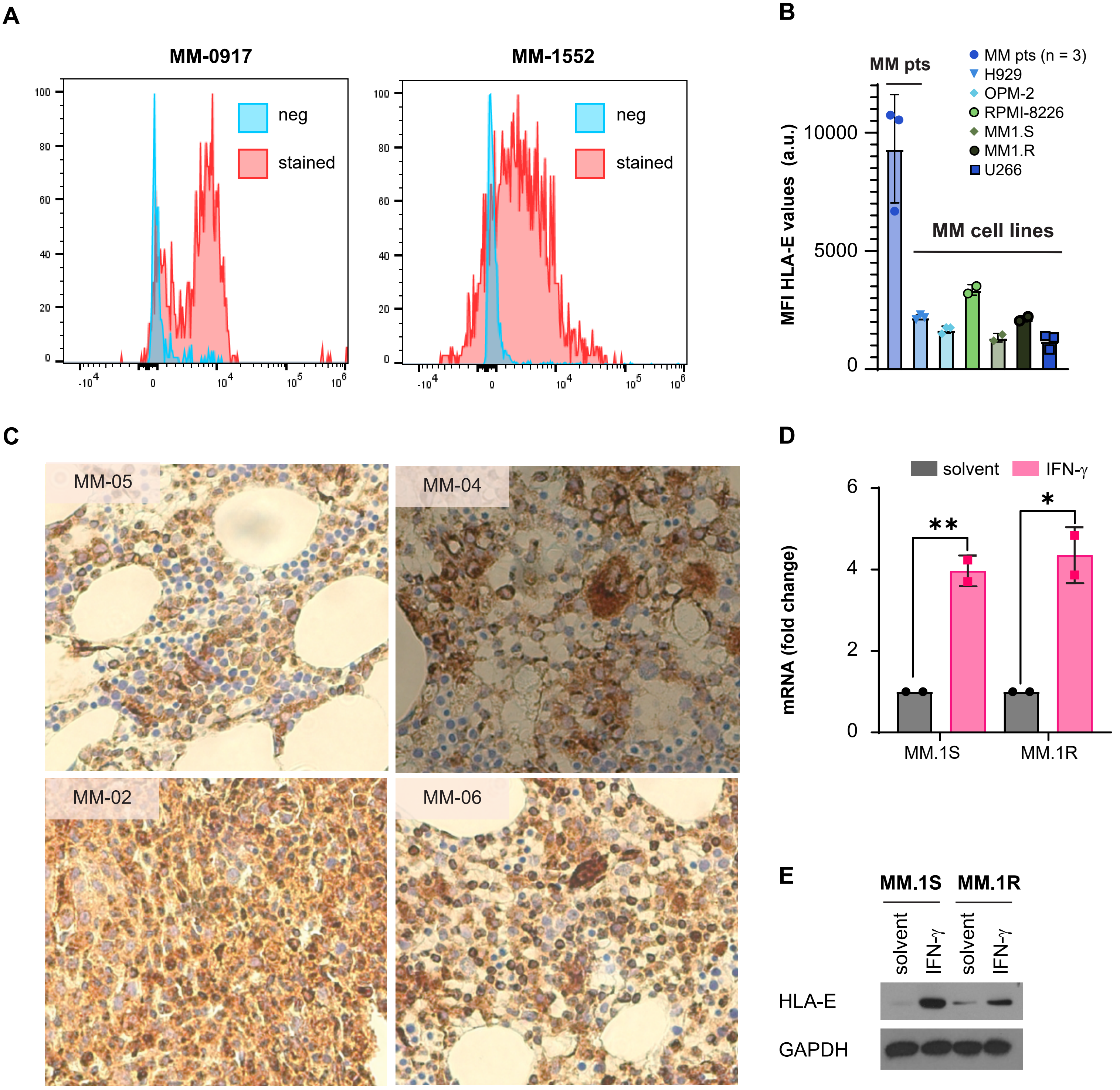

Figure S1. HLA-E expression in MM. Related to Figure 1.

(A) Flow cytometry staining for HLA-E in patients with Multiple Myeloma (MM).
(B) Mean fluorescence intensity (MFI) levels of HLA-E in n = 3 patients with MM and in a panel of MM cell lines.
(C) Immunohistochemistry staining for HLA-E (20X) in newly diagnosed with MM (n = 4).
(D) HLA-E mRNA fold change in MM.1S and MM.1R cells treated with solvent or IFN-γ 1 ng/mL for 24 hours. n = 2, t test, two-tailed; MM.1S p = 0.0080, **; MM.1R p = 0.0203, *.
(E) Western blot analysis for HLA-E and GAPDH in MM.1S and MM.1R treated with solvent or IFN-γ 1 ng/mL for 24 hours.

Figure S2

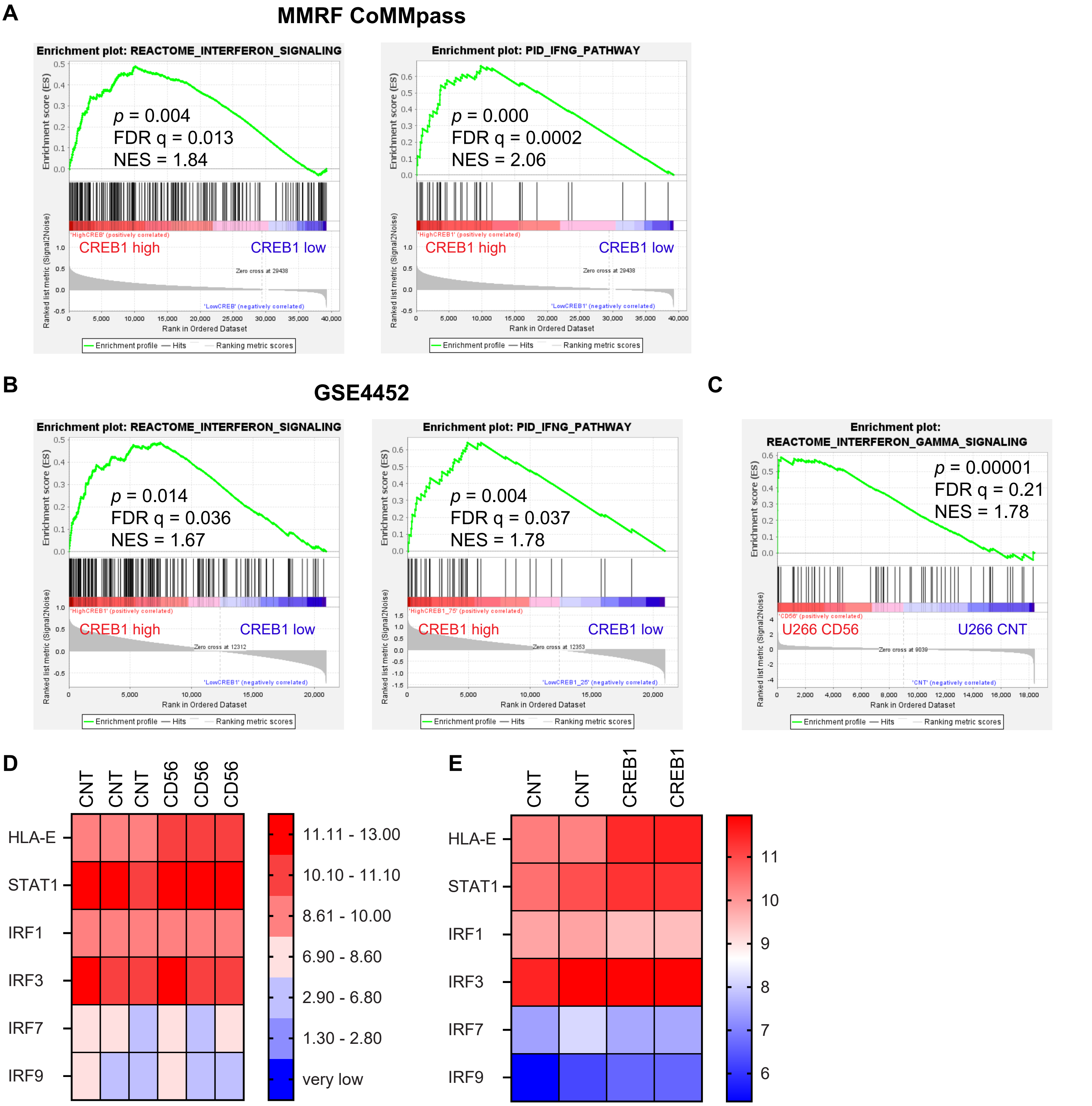

Figure S2. In silico analysis for IFN-γ-related pathways. Related to Figure 2.

(A-B) Enrichment plots of IFN-driven pathways from C2_CP_REACTOME_INTERFERON_SIGNALING and C2_CP_PID_IFNG_PATHWAY in the MMRF CoMMpass dataset (**A**) or GSE4452 dataset (**B**). Patients are divided based on median cutoff of CREB1 expression. NES, normalized enrichment score. FDR, false discovery rate.
(C) Enrichment plot of C2_CP_REACTOME INTERFERON_GAMMA_SIGNALING gene set in U266 control cells (CNT) or U266 overexpressing CD56. n = 3 replicates.
(D) Heat map with the genes of C2_CP_REACTOME INTERFERON_GAMMA_SIGNALING in U266 control cells (CNT) or U266 overexpressing CD56. n = 3 replicates.
(E) Heat map with the genes of C2_CP_REACTOME INTERFERON_GAMMA_SIGNALING in U266 control cells (CNT) or U266 overexpressing CREB1. n = 2 replicates.

Figure S3

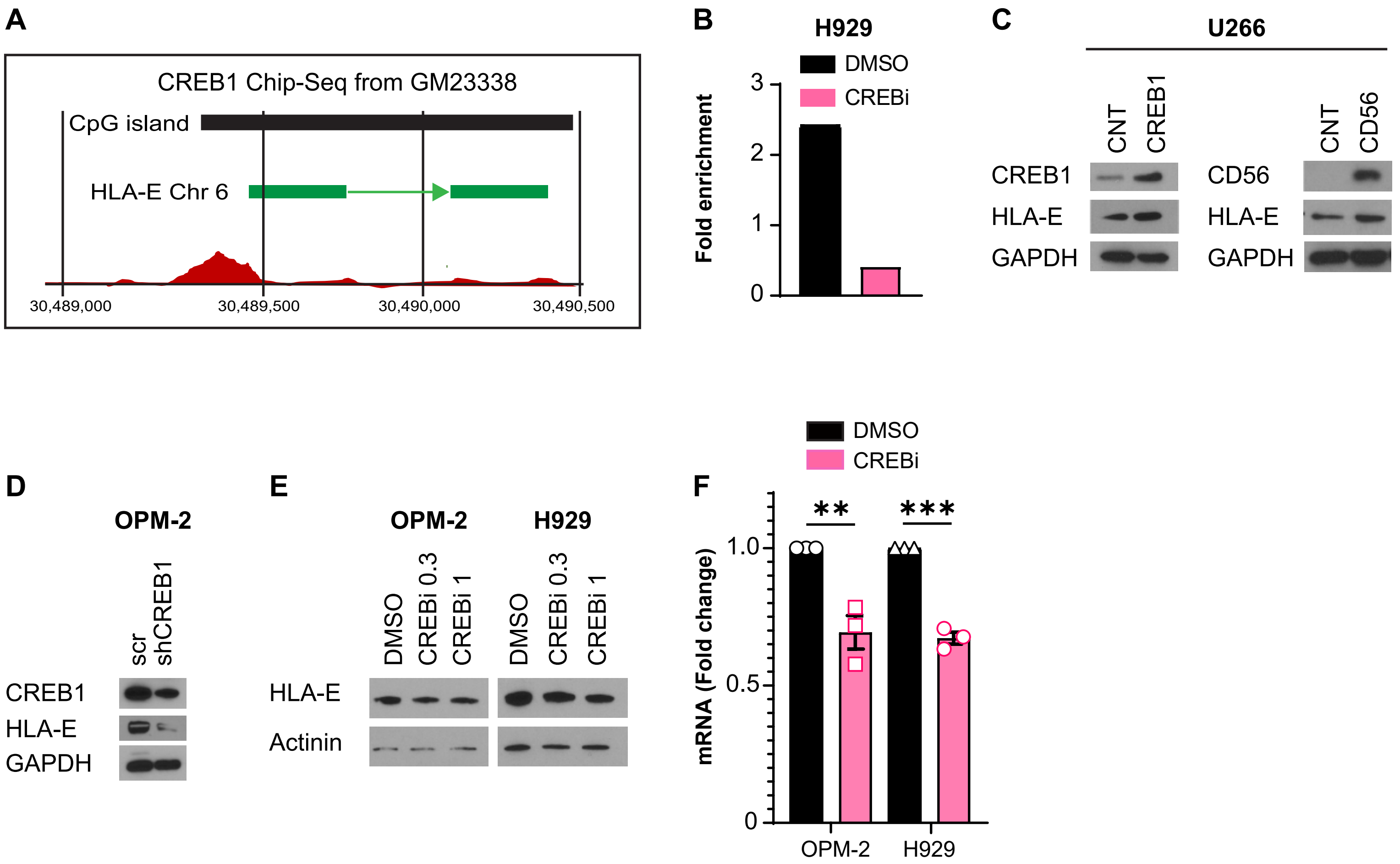

Figure S3. HLA-E modulation by CD56 or CREB1. Related to Figure 3.

(A) Analysis of CREB1 binding in the human B-cell line GM12878 by ChIP-seq from the ENCODE Project. Shown is the mapping track of sequence reads at the HLA-E locus on chromosome 6.
(B) Fold enrichment for HLA-E promoter binding in control and H929 cells treated with 666-15 (CREBi) 2 μM for 24 hours.
(C) Western blot analysis for HLA-E in U266 control cells (CNT) or cells overexpressing CREB1 (Left) or in U266 control cells (CNT) or cells overexpressing CD56 (Right) at 48 hours after transfection. GAPDH is used as normalizer; CD56 and CREB1 are shown to confirm overexpression.
(D) Western blot analysis for HLA-E, CREB1, and GAPDH in OPM-2 control cells (scr) or silenced for CREB1 (shCREB1).
(E) Western blot analysis for HLA-E and actinin in OPM-2 and H929 cells treated with DMSO, 666-15 (CREBi) 0.3 μM, and CREBi 1 μM for 72 hours.
(F) HLA-E mRNA fold change in OPM-2 and H929 cells treated with DMSO and 666-15 (CREBi) 0.3 μM for 24 hours. n = 3, t test, two-tailed; OPM-2 $p = 0.0074$, **; and H929 $p = 0.0001$, ***.

Figure S4

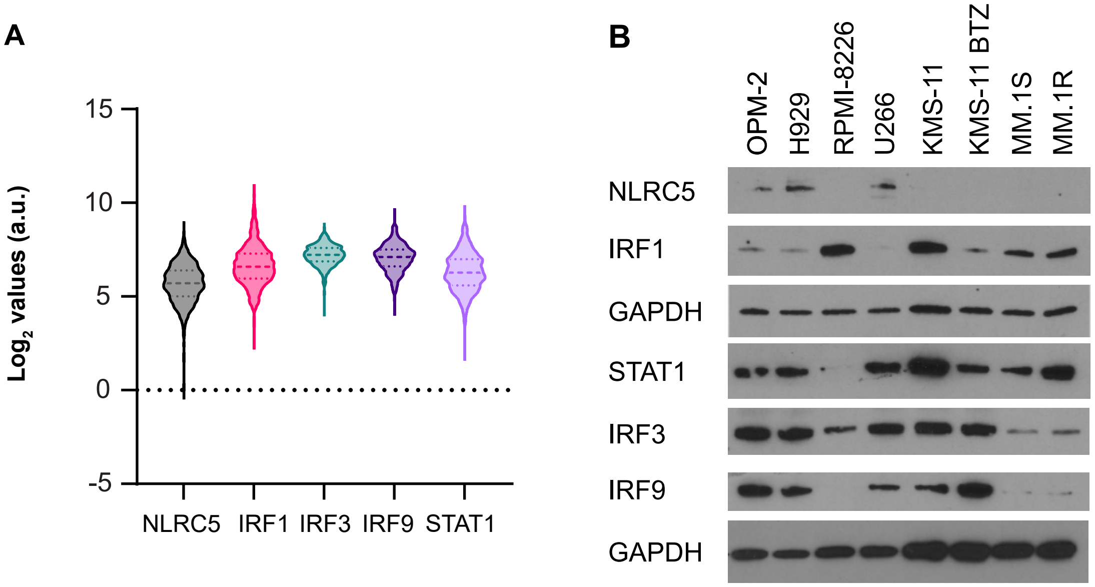

Figure S4. STAT1 pathway in MM. Related to Figure 3.

(A) NLRC5, IRF1, IRF3, IRF9, and STAT1 $\log_2$ expression values in n = 809 patients with MM from the MMRF CoMMpass dataset. Dashed lines indicate the median values; dotted lines represent the 25th and 75th percentile.
(B) Western blot analysis for NLRC5, IRF1, IRF3, IRF9, and STAT1 in a panel of MM cell lines. The same lysates were run in duplicates. GAPDH was re-blotted on each membrane.

Figure S5

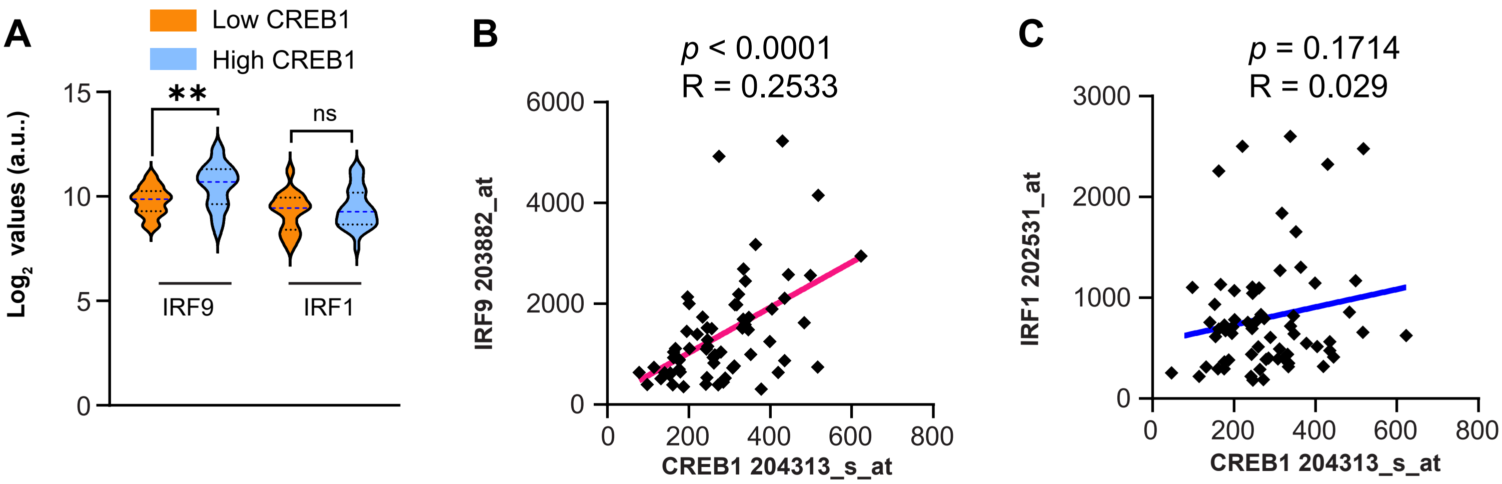

Figure S5. *In silico* correlation of CREB1 with IRF9 or IRF1 in the GSE4452 dataset. Related to Figure 3.

(A) IRF9 and IRF1 log_2 mRNA values in patients with low or high CREB1 expression based on median cutoff of CREB1 expression. IRF9 $p = 0.0012$, **; IRF1 p = not significant, ns. Dashed blue lines indicate median values; dotted lines represent the 25th and 75th percentile.
(B) Regression studies to correlate IRF9 (probe 203882_at) as dependent variable to CREB1 (probe 204313_at). $p < 0.0001$; R = 0.2533.
(C) Regression studies to correlate IRF1 (probe 202531_at) as dependent variable to CREB1 (probe 204313_at). $p = 0.1714$, ns; R = 0.029.

Figure S6

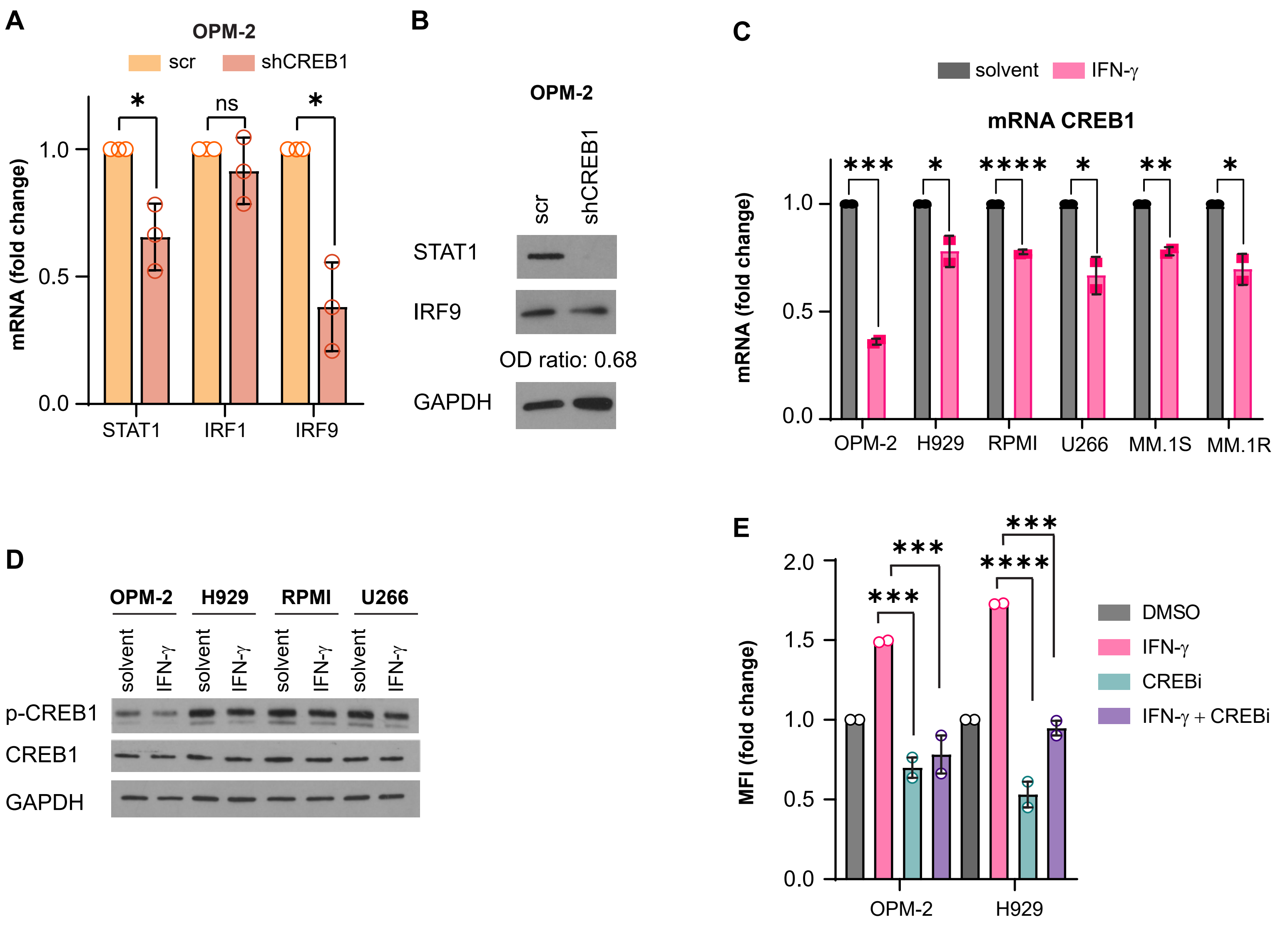

Figure S6. CREB1 and STAT1 regulation. Related to Figure 3.

(A) STAT1, IRF1, and IRF9 mRNA fold change in OPM-2 scrambled cells (scr) or OPM-2 cells silenced for CREB1 (shCREB1). n = 3, t test, two-tailed; STAT1 $p = 0.038$, *; IRF1 $p = 0.32$, ns; IRF9 $p = 0.017$, *.
(B) Western blot analysis for STAT1, IRF9, and GAPDH of OPM-2 scrambled cells (scr) and OPM-2 cells silenced for CREB1 (shCREB1). Optical densities (OD) ratio for IRF9 normalized to GAPDH is reported in the figure.
(C) CREB1 mRNA fold change in OPM-2, H929, RPMI-8226, U266, MM.1S, and MM.1R treated with solvent or IFN-γ 1 ng/mL for 24 hours. n = 2; t test, two-tailed; *p* values are 0.0002, ***; 0.05, *; 0.00002, ****; 0.03, *; 0.003, **; and 0.027, * in the order presented.
(D) Western blot analysis for phospho-CREB1, total CREB1, and GAPDH in OPM-2, H929, RPMI-8226, and U266 cells treated with solvent or IFN-γ 1 ng/mL for 24 hours.
(E) HLA-E MFI fold change in OPM-2 and H929 treated with DMSO, IFN-γ 1 ng/mL, 666-15 (CREBi) 1 μM, and the combination of IFN-γ + CREBi. n = 2, t test, two tailed; OPM-2 cells: IFN-γ versus CREBi $p = 0.0002$, ***; IFN-γ versus IFN-γ + CREBi $p = 0.0004$, ***. H929 cells: IFN-γ versus CREBi $p < 0.0001$, ****; IFN-γ versus IFN-γ + CREBi $p = 0.0002$, ***.

Figure S7

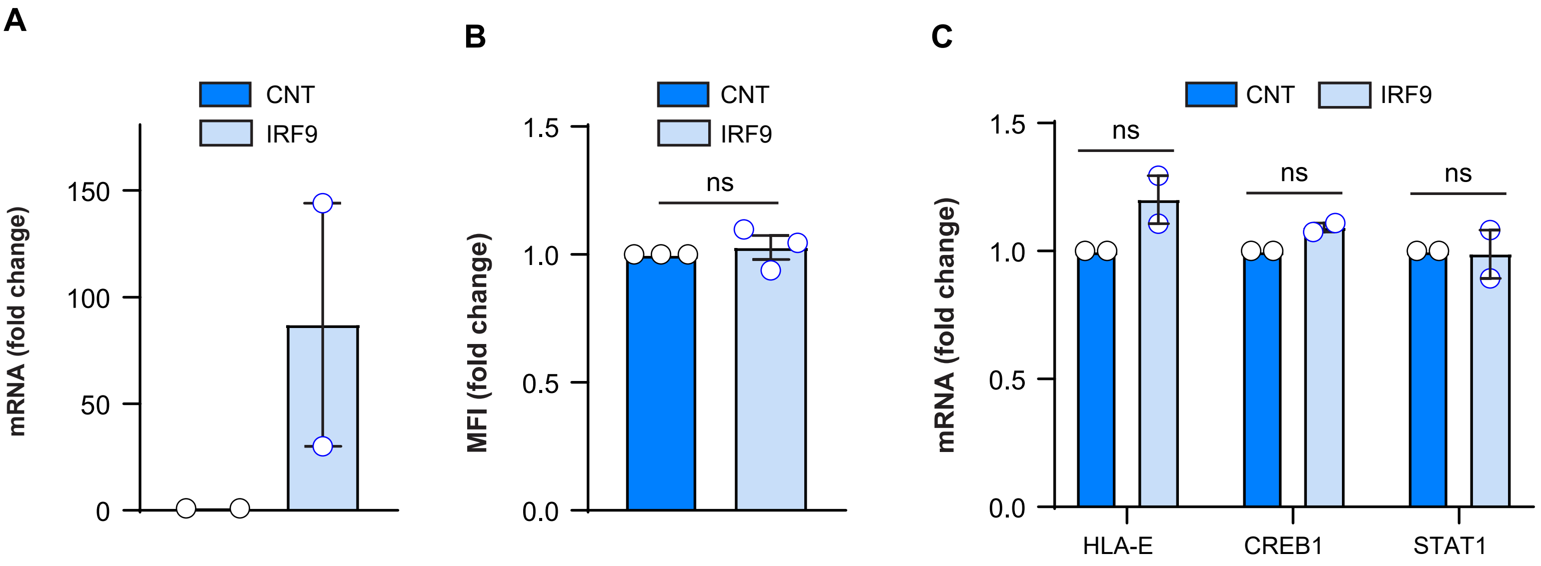

Figure S7. IRF9 overexpression in MM. Related to Figure 3.

(A) IRF9 mRNA fold change in U266 control cells (CNT) or U266 cells overexpressing IRF9.
(B) HLA-E MFI fold change in U266 control cells (CNT) or U266 cells overexpressing IRF9. $p = 0.59$, ns.
(C) HLA-E, CREB1, and STAT1 mRNA fold change in U266 control cells (CNT) or U266 cells overexpressing IRF9. n = 2; t test, two-tailed; HLA-E $p = 0.16$, ns; CREB1 $p = 0.3$, ns; STAT1 $p = 0.90$, ns.

Figure S8

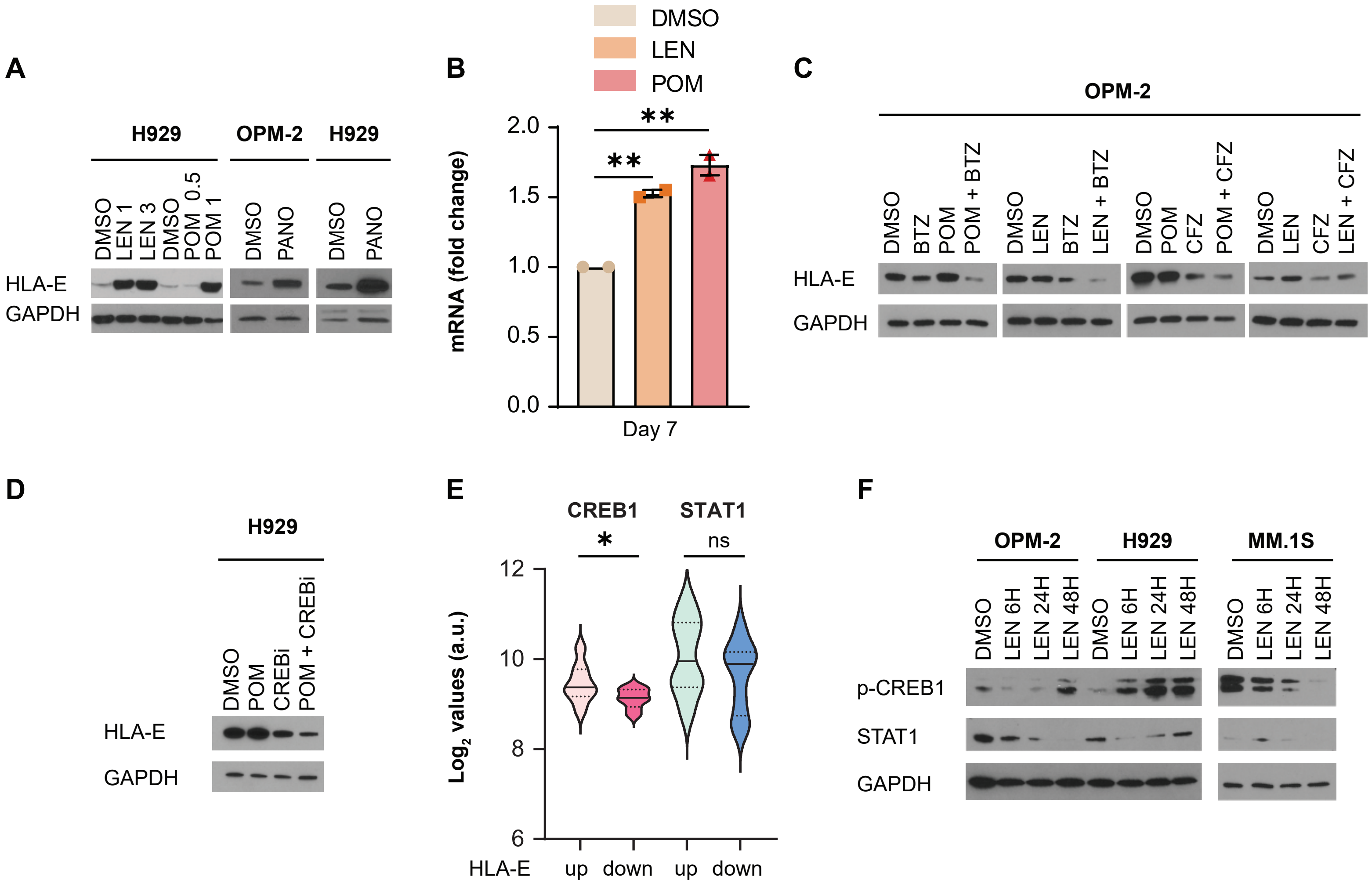

Figure S8. Regulation of HLA-E expression by anti-MM drugs and CREB1 inhibitors. Related to Figure 4.

(A) Western blot analysis for HLA-E and GAPDH in H929 cells treated with DMSO, LEN 1 μM, LEN 3 μM, POM 0.5 μM, or POM 1 μM for 72 hours; OPM-2 and H929 cells treated with DMSO and PANO 100 nM for 24 hours.
(B) HLA-E mRNA fold change in OPM-2 cells treated with DMSO, LEN 0.1 μM, or POM 1 μM for 7 days. n = 2, t test, two-tailed. DMSO versus LEN $p = 0.0024$, **; DMSO versus POM $p = 0.0098$, **.
(C) Western blot analysis for HLA-E and GAPDH in OPM-2 cells treated with DMSO, bortezomib (BTZ) 5 nM, pomalidomide (POM) 1 μM, lenalidomide (LEN) 1 μM, or carfilzomib (CFZ) 5 nM either as single agents or in combination for 24 hours.
(D) Western blot analysis for HLA-E and GAPDH in H929 cells treated with DMSO, pomalidomide (POM) 1 μM, 666-15 (CREBi) 0.3 μM and combination of POM and CREBi (POM + CREBi) for 72 hours.
(E) CREB1 (probe 877_at) and STAT1 (probe 32859_at) $\log_2$ mRNA expression values in n = 8 with HLA-E increased post-lenalidomide (up) or decreased post-lenalidomide (down). CREB1 $p = 0.05$, *; STAT1 $p = 0.27$, ns. Solid black lines indicate the median values; dotted black lines represent the 25th and 75th percentiles.
(F) Western blot analysis for phospho-CREB1, STAT1, and GAPDH in OPM-2, H929, and MM.1S cells treated with DMSO or lenalidomide (LEN) 1 μM for 6, 24, and 48 hours.

Figure S9

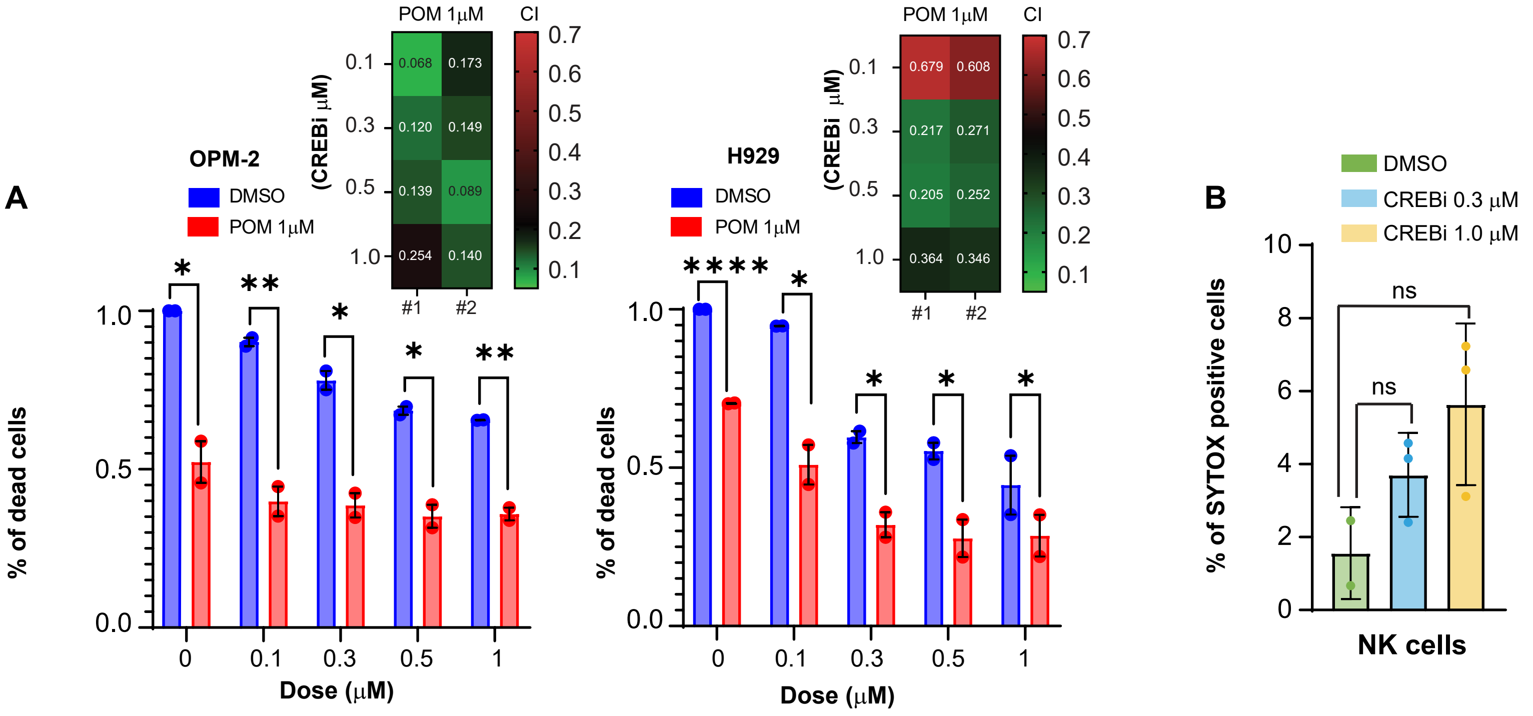

Figure S9. Cytotoxic effects of CREB1 inhibitors and/or IMiDs on myeloma cells and natural killer (NK) cells. Related to Figure 5.

(A) Viability analysis by MTT assay in H929 and OPM-2 cells treated with DMSO, pomalidomide (POM) 1 μM and 666-15 (CREBi) at 0.1-3 μM for 72 hours. n = 2, t test, two-tailed. OPM-2: POM versus DMSO p = 0.018, *; POM versus CREBi 0.1 μM p = 0.009, **; POM versus CREBi 0.3 μM p = 0.014, *; POM versus CREBi 0.5 μM p = 0.012, *; POM versus CREBi 1 μM p = 0.004, **. H929: POM versus DMSO p < 0.0001, ****; POM versus CREBi 0.1 μM p = 0.019, *; POM versus CREBi 0.3 μM p = 0.024, *; POM versus CREBi 0.5 μM p = 0.05, *; POM versus CREBi 1 μM p = 0.029, *. Inserts show heat map of the synergic effects calculated by Chou-Talalay. CI stays for combination index.
(B) Cell death analysis by SYTOX staining of NK cells isolated from 3 healthy donors and treated for 48 hours with DMSO, 666-15 (CREBi) 0.3 μM, or CREBi 1 μM.

Figure S10

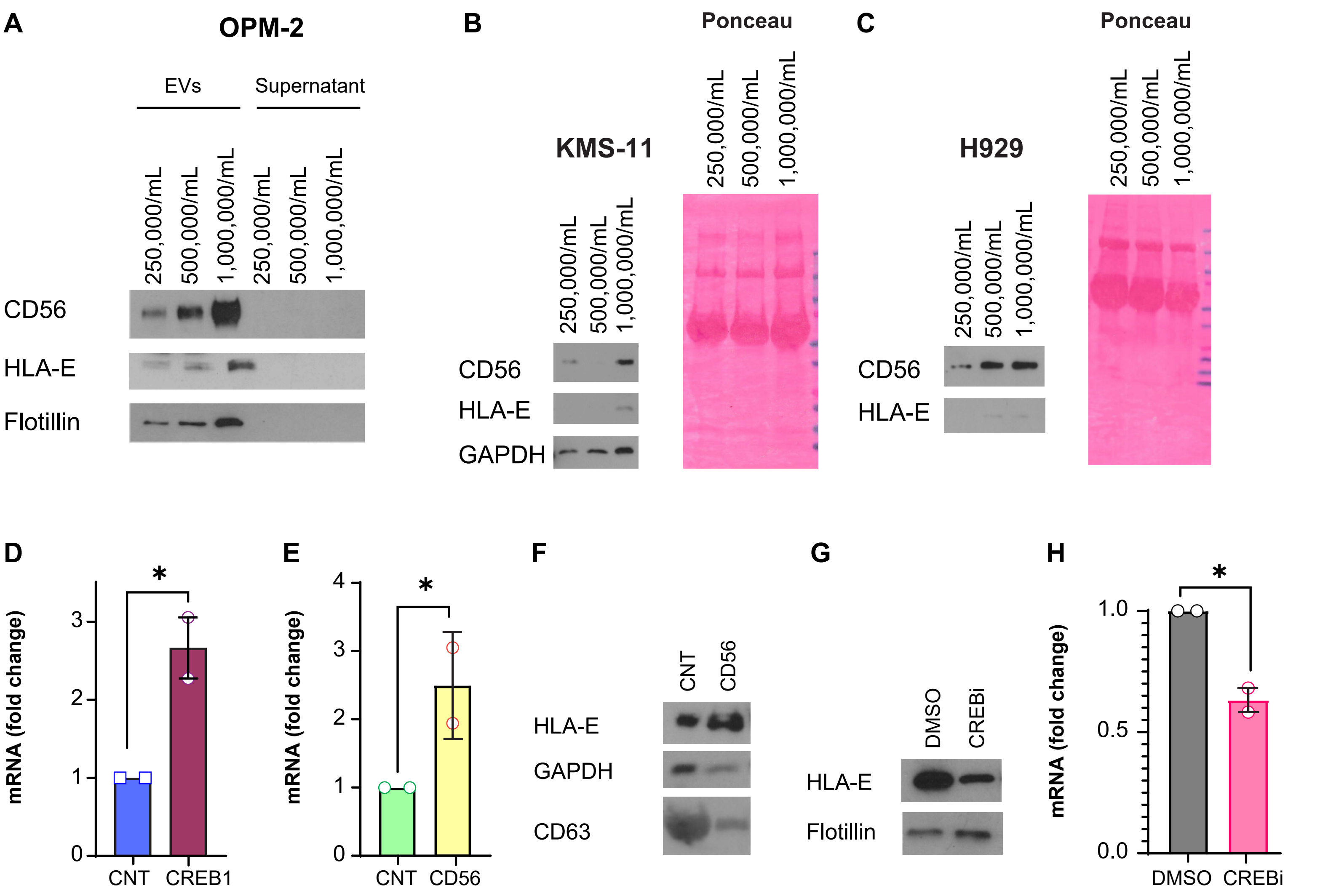

Figure S10. HLA-E and its presence in extracellular vesicles (EVs). Related to Discussion.

(A-C) EVs isolated from OPM-2, KMS-11, and H929 plated at different cellular concentrations. Western blot and Ponceau S staining are shown.

(D) HLA-E mRNA fold change in extracellular vesicles (EVs) isolated from U266 control cells (CNT) or U266 cells overexpressing CREB1. n = 2, t test, two-tailed; $p = 0.05$, *.

(E) HLA-E mRNA fold change in EVs isolated from U266 control cells (CNT) or U266 cells overexpressing CD56. n = 2, t test, two-tailed; $p = 0.047$, *.

(F) Western blot analysis for HLA-E, GAPDH, and CD63 using protein content isolated from EVs derived from U266 control cells (CNT) or U266 cells overexpressing CD56.

(G) Western blot analysis for HLA-E and flotillin using protein content isolated from EVs derived from OPM-2 cells treated with DMSO or 666-15 (CREBi) 0.3 μM for 48 hours.

(H) HLA-E mRNA fold change in EVs isolated from OPM-2 cells treated with DMSO or 666-15 (CREBi) 0.3 μM for 48 hours. n = 2, t test, two-tailed; p = 0.0177, *.
